# Mitochondrial genome recombination in somatic hybrids of *Solanum commersonii* and *S. tuberosum*

**DOI:** 10.1038/s41598-022-12661-z

**Published:** 2022-05-23

**Authors:** Kwang-Soo Cho, Hyun-Oh Lee, Sang-Choon Lee, Hyun-Jin Park, Jin-Hee Seo, Ji-Hong Cho, Young-Eun Park, Jang-Gyu Choi, Tae-Jin Yang

**Affiliations:** 1 Department of Southern Area Crop Science, National Institute of Crop Science, Rural Development Administration, Miryang, 50424 Republic of Korea; 2 Department of Agriculture, Forestry and Bioresources, Plant Genomics and Breeding Institute, College of Agriculture and Life Sciences, Seoul National University, 1 Gwanak-ro, Gwanak-gu, Seoul, 08826 Republic of Korea; 3 Highland Agriculture Research Institute, National Institute of Crop Science, Rural Development Administration, Pyeongchang, 25342 Republic of Korea; 4 Phyzen Genomics Institute, Baekgoong Plaza 1, Bundang-gu, Seongnam, 13558 Republic of Korea

**Keywords:** Evolution, Plant sciences

## Abstract

Interspecific somatic hybridization has been performed in potato breeding experiments to increase plant resistance against biotic and abiotic stress conditions. We analyzed the mitochondrial and plastid genomes and 45S nuclear ribosomal DNA (45S rDNA) for the cultivated potato (*S. tuberosum*, St), wild potato (*S. commersonii*, Sc), and their somatic hybrid (StSc). Complex genome components and structure, such as the hybrid form of 45S rDNA in StSc, unique plastome in Sc, and recombinant mitogenome were identified. However, the mitogenome exhibited dynamic multipartite structures in both species as well as in the somatic hybrid. In St, the mitogenome is 756,058 bp and is composed of five subgenomes ranging from 297,014 to 49,171 bp. In Sc, it is 552,103 bp long and is composed of two sub-genomes of 338,427 and 213,676 bp length. StSc has 447,645 bp long mitogenome with two subgenomes of length 398,439 and 49,206 bp. The mitogenome structure exhibited dynamic recombination mediated by tandem repeats; however, it contained highly conserved genes in the three species. Among the 35 protein-coding genes of the StSc mitogenome, 21 were identical for all the three species, and 12 and 2 were unique in Sc and St, respectively. The recombinant mitogenome might be derived from homologous recombination between both species during somatic hybrid development.

## Introduction

Potato (*Solanum tuberosum*) belongs to Solanaceae and is the fourth important food crop for human consumption worldwide. Plant cytoplasmic genomes, such as plastid (plastomes) and mitochondrial (mitogenomes) genomes, are maternally inherited and are usually present in high copy numbers in plant cells^1^. The mitochondrion independently duplicates its genome and governs the cell energy supply and plant development^2^. The mitogenome exists as a mixture of variable sizes mediated by recombination between tandem repeat sequences^3^, while plastomes are highly conserved in their variation and size^4^. High levels of recombination and foreign DNA integration result in most of the size variation in mitogenomes, and variable forms of mitochondrial gene transcripts occur due to multiple transcript initiation and termination sites, trans-splicing, and RNA editing^4–7^. The intraspecific structural variation of the mitogenome has been found to be extraordinarily high in plant species, such as the multipartite forms in *Silene* species^8^.

While cytoplasmic genetic elements are maternally inherited in most plants^9,10^, their inheritance patterns are quite different in somatic hybrids generated by conducting the protoplast fusion. Somatic hybrids carry plastomes derived from one of the two parents without sequence variation or rearrangement^11,12^. However, mitogenome maintenance has not yet been reported in somatic hybrids. A previous study suggested that mitogenomes might be presented as fused forms by finding a novel fingerprinting band pattern in the somatic hybrid progenies of *Solanum melongena* cv. Black Beauty and *Solanum torvum*^13^.

Complete mitogenome sequences have been released from over 11,354 species of animals, fungi, plants, and protists (Organelle Genome Resources, https://www.ncbi.nlm.nih.gov/genome/organelle/), which has facilitated research on the genetic and evolutionary nature of mitogenomes^14–16^. Mitogenome sequences were released for crop species in Solanaceae including pepper (*Capsicum annum*), potato (*S. tuberosum*), and tomato (*S. lycopersicum*)^17–19^, and mitogenome structures and phylogenetic relationships among these species have been revealed. Previously, we reported the complete mitogenome sequences for St and Sc; however, a detailed genome analysis was not conducted^18,20^.

Somatic hybridization mediated by protoplast fusion can be a useful breeding tool for introducing valuable traits from related species^21–23^. Favorable traits such as disease resistance^24,25^ and drought tolerance^26^ were transmitted to the cultivated potato from wild species. Similar approaches have been applied to develop potato somatic hybrids with enhanced disease resistance using wild potato species^27–31^. The organelle genome structures in somatic hybrids have been investigated in these studies. Conserved intact plastomes were delivered to the somatic hybrid from either of the two parents. Novel mitogenomes have been identified via non-random rearrangement in somatic hybrids^21,29,32^. Previous studies have found putative rearrangement hotspots and specific regions preferentially inherited or eliminated. In addition, Lossl et al.^33^ speculated that non-random mitochondrial rearrangement determining mitogenome types in somatic hybrids might be related to yield components in potatoes. However, these studies did not provide sequence-level evidence for mitogenome recombination events in somatic hybrids.

In this study, we characterized plastome, mitogenomes, and nuclear ribosomal DNA (nrDNA) of tetraploid potato (*S. tuberosum*), wild diploid potato (*S. commersonii*), and their somatic hybrid. The comparative analysis revealed their inheritance pattern and exhibited dynamic multipartite structural variation mediated by recombination events that share unique genes from both species in the somatic hybrid mitogenome.

## Results

### Complete mitochondrial genome assembly

The mitogenomes of St, Sc, and StSc were assembled into five to two subgenomes through de novo assembly using 5.3 to 6.6 Gb PE reads. Each assembly was validated by conducting PCR analysis and sequencing (Tables S1 and S2, Fig. S1). The St mitogenome size was 756,058 bp, and it was composed of five circular subgenomes of lengths 49,230 to 297,014 bp. The total number of non-redundant genes was 78, consisting of 37 PCGs, 19 ORFs, 3 rRNAs, and 19 tRNAs (Table 1, Fig. S2A). The Sc mitogenome was 552,103 bp in size with two subgenomes (338,427 and 213,676 bp). The total number of non-redundant genes was 77, consisting of 37 PCGs, 20 ORFs, 3 rRNAs, and 17 tRNAs (Table 1, Fig. S2B). The StSc mitogenomes were 447,645 bp in size with a major circular DNA of 398,439 bp and a minor subgenome of 49,206 bp. The total number of non-redundant genes was 77, consisting of 37 PCGs, 20 ORFs, 3 rRNAs, and 17 tRNAs (Table 1, Fig. S2C).

**Table 1:** Mitogenome features of *S. tuberosum, S. commersonii*, and their somatic hybrid.

Scientific name	Somatic hybrid		*S. commersonii*		*S. tuberosum*				
Subgenomes	1	2	1	2	1	2	3	4	5
Genome length (bp)	398,439	49,206	338,427	213,676	297,014	247,843	112,800	49,171	49,230
No. of total genes (non-redundant)	77		77		78				
No. of protein coding genes	37		37		37				
No. of hypothetical genes	20		20		19				
No. of ribosomal RNA genes	3		3		3				
No. of transfer RNA genes	17		17		19				
No. of total genes by subgenome	68	14	64	48	49	30	22	12	13
No. of protein coding genes	32	6	31	19	20	19	12	5	6
No. of hypothetical genes	19	3	18	14	15	0	4	2	2
No. of ribosomal RNA genes	3	0	2	3	3	3	1	0	0
No. of transfer RNA genes	14	5	13	12	11	8	5	5	5
Proportion of MTPT (%)	2.9	8.0	3.1	4.0	1.5	1.0	5.0	4.3	8.0
Proportion of NUMT (%)	16.1	17.4	16.3	10.1	24.1	17.5	20.0	57.7	17.2
Proportion of repeat sequence (%)	21.3	4.8	25.9	5.7	19.4	15.2	5.5	2.2	4.9
GenBank accession nos	MF989958	MF989959	MF989960	MF989961	MF989953	MF989954	MF989955	MF989956	MF989957

*MTPT* mitochondrial plastid DNA, *NUMT* nuclear mitochondrial DNA.

A total of 71 genes were shared among the three mitogenomes. Some genes were unique in each mitogenome: four ORFs (*orf131, orf 190, orf 240,* and *orf 279*), and three tRNAs (*trnI*-GAU, *trnL*-CAA, *and trnV*-GAC) were unique in the St mitogenome; five ORFs (*orf109d, orf111, orf140, orf185, orf240*) and one tRNA (*trnfM-*CAT) were unique in the Sc genome; and five ORFs (*orf111, orf127, orf131, orf140, orf185*) and one tRNA (*trnV-*GAC) were unique in the StSc mitogenome (Table 2).

**Table 2:** Common and unique mitogenome genes of a somatic hybrid and its parent species.

Group of genes	Common genes	Unique genes		
		*Somatic hybrid*	*S. commersonii*	*S. tuberosum*
Complex I	*nad1, 2, 3, 4, 4L, 5, 6, 7, 9*			
Complex II	*sdh3, sdh4*			
Complex III	*cob*			
Complex IV	*cox1, 2, 3*			
Complex V	*atp1, 4, 6, 8*(*orfB*)*, 9*			
Cytochrome c biogenesis	*ccmB, ccmC, ccmFc, ccmFn*			
Large subunit ribosomal proteins	*rpl2, 5, 10, 16*			
Small subunit ribosomal proteins	*rps1, 3, 4, 10, 12, 13, 19*			
Maturase	*matR*			
Transferase	*mttB *(*orfX*)			
Ribosomal RNAs	*rrn5, rrn18, rrn26*			
Hypothetical genes	*orf100, 101, 102, 103, 104, 105, 108, 109, 110, 122, 123, 125, 138, 169, 261*	*orf111, 127, 131, 140, 185*	*orf109d, 111, 140, 185, 240*	*orf131, 190, 240, 279*
Transfer RNAs	*trnC-GCA, trnD-GUC, trnE-UUC, trnF-GAA, trnG-GCC, trnH-GUG, trnK-UUU, trnM-CAU, trnN-GUU, trnP-UGG, trnQ-UUG, trnS-GCU, trnS-GGA, trnS-UGA, trnW-CCA, trnY-GUA*	*trnV-GAC*	*trnfM-CAT*	*trnI-GAU, trnL-CAA, trnV-GAC*
No. of genes	71	6	6	7

### Mitogenome homologs in plastome and nuclear genome

Mitochondrial plastid DNA (MTPT) has been reported in various plants, such as *Amborella trichopoda*, *Zea mays* (maize), and *Cynanchum wilfordii*^34–36^. The degree of MTPT was examined by sequence comparison with the *S. tuberosum* plastome sequence (GenBank accession No. no. KM489056)^37^. Consequently, the St, Sc, and StSc mitogenomes were approximately 1.0–8.0%, 2.9–8.0%, and 3.1–4.0% considered as MTPT, respectively. Overall, approximately 1.0–8.0% were identified as MTPT (Table 1, Fig. S2).

Further, nuclear mitochondrial DNA (NUMT) has also been reported in various plants, such as *Arabidopsis thaliana* and *Cucumis sativus* (cucumber)^38,39^. NUMT was identified by sequence comparison with the *S. tuberosum* nuclear genome sequence (SolTub_3.0, https://www.ncbi.nlm.nih.gov/assembly/GCF_000226075.1/). Consequently, the St, Sc, and StSc mitogenomes were approximately 17.2–57.7%, 16.1–17.4%, and 10.1–16.3%, respectively, which were considered to be derived from or transferred to nuclear genomes accordingly. Overall, approximately 10.7–57.7% was identified as NUMTs. A total of 57.7% was identified in St subgenome 4, which has a very small genome size (Table 1, Fig. S2).

### Homologous recombination mediated by large repeats in mitogenomes

**Figure 1:**
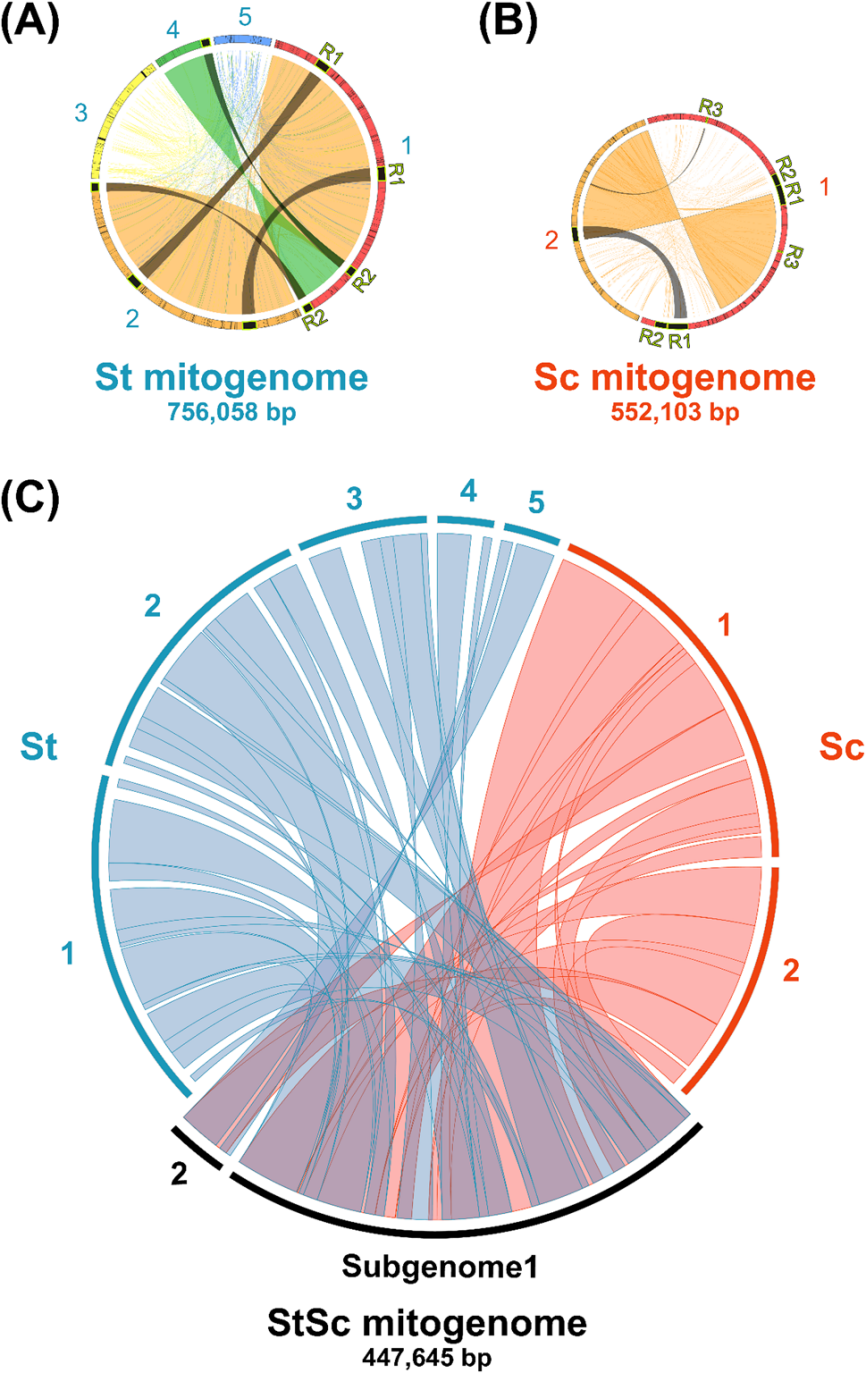
Chord diagram of three *Solanum* mitogenomes. (**A**–**C**) represent the homologous regions of the subgenomes. R1 to R3 represent the large repeats that might cause homologous recombination among the corresponding subgenomes. St*: S. tuberosum* accession no. PT56, Sc: *S. commersonii* accession no. Lz3.2, StSc: somatic hybrid accession no. HA06-9.

Homologous recombination (HR) can be mediated by repeat sequences in St, Sc, and StSc mitogenomes. The St, Sc, and StSc mitogenomes accounted for approximately 2.2–19.4%, 4.8–21.3%, and 5.7–25.9% of repeat sequences in which the repeat ratio was also positively correlated with the subgenome size (Table 1, Figs. 1 and S2). The five St subgenomes exhibited diverse numbers of dispersed repeats: 300 (mitogenome coverage: 19.4%), 211 (15.2%), 41 (5.5%), 18 (2.2%), and 39 (4.9%) in each subgenome (Tables 1 and S5, Figs. 1A and S2A). The two Sc subgenomes included 460 (25.9%) and 198 dispersed repeats (15.2%) (Tables 1 and S5, Figs. 1B and S2B). Further, the two StSc subgenomes contained 480 (21.3%) and 39 (4.8%) dispersed repeats (Tables 1 and S5, Fig. 1C and S2C). In contrast, tandem repeats were selected with adjacent sequences of at least two copies and up to 50 bp. The St, Sc, and StSc mitogenomes had only 17, 20, and 16 tandem repeats, respectively (Table S6).

Two large repeats (more than 1 kb) were identified in the St subgenome 1. R1 was 11,916 bp, and R2 was 7500 bp. In contrast, St subgenome 2 had only R1, and subgenome 3 had only 1589 bp of R3. Similarly, the R1 sequence co-existed in St subgenomes 1 and 2. The R2 repeat is shared between subgenomes 1 and 4 (Table S5, Figs. 1 and S2), which might contribute to the HR between different subgenomes. The Sc mitogenomes had two multipartite structures, in which three large repeats of more than 1 kb were identified (R1: 16,857 bp, R2: 10,094 bp, and R3: 1024 bp), which might contribute to recombination events between subgenomes (Table S5, Figs. 1 and S2). The StSc mitogenomes contain four large repeats (more than 1 kb) (R1, 11,916 bp; R2, 11,846 bp; R3, 1643 bp; and R4, 1024 bp) that might contribute to subgenome reshuffling (Table S5, Figs. 1 and S2).

### Confirmation of the somatic hybrid in mitochondria and nuclear genomes

**Figure 2:**
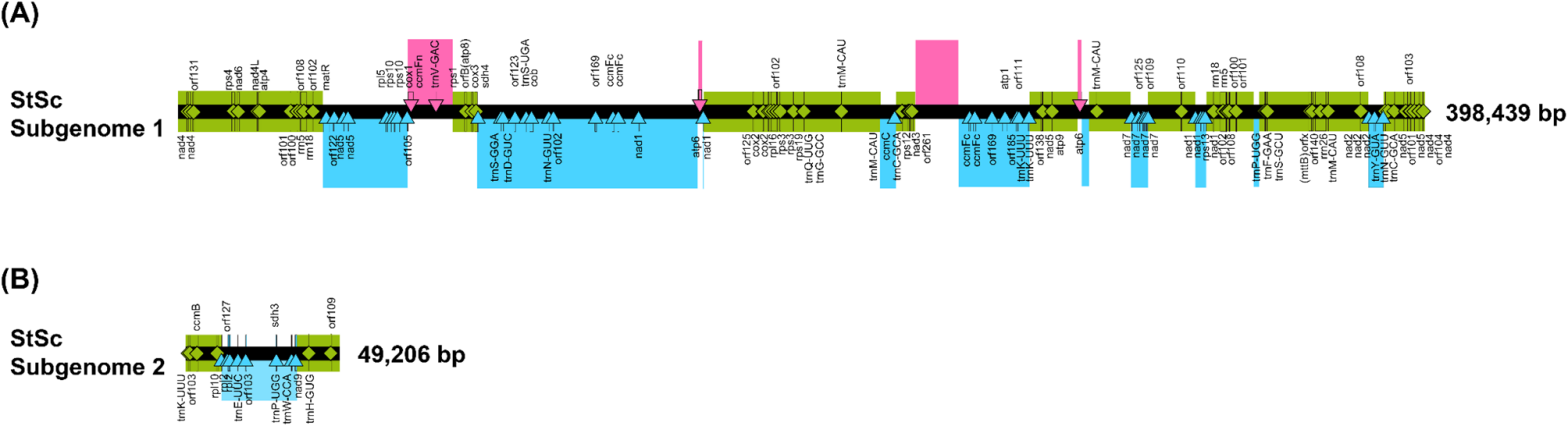
The origin of mitogenome recombination block in somatic hybrid (StSc) (**A**) Subgenome 1 of somatic hybrid mitogenome (**B**) Subgenome 2 of somatic hybrid mitogenome. The pink and sky-blue triangles on the black middle line indicate genes derived from *S. tuberosum* and *S. commersonii*, respectively. The green diamond boxes indicate genes of unknown origin.

We compared plastomes, mitogenomes, and nrDNAs among St, Sc, and StSc genomes. The StSc plastome was identical to Sc plastome^37^. Meanwhile, the StSc mitogenome shows a complicated structure with unique genes derived from both species (Table S3, Fig. 2). Among 71 common genes, 21 PCGs (*nad3, nad4, nad4L, nad5, nad6, sdh3, cox2, cox3, atp1, atp4, atp8, atp9, ccmB, rps3, rps4, rps12, rps13, rpl5, rpl10, rpl16,* and *mttB*) were found identical across the three mitogenomes (denoted as green boxes on Fig. 2) and their origin in the StSc genome could not be determined; 12 PCGs (*nad1, nad2, nad7, nad9, sdh4, cob, cox1, ccmC, ccmFc, rps10, rpl2,* and *matR*) were found identical with Sc (represented as sky-blue boxes in Fig. 2) and 2 PCGs (*atp6* and *ccmFN*) were identical with St (pink boxes in Fig. 2). Therefore, it is likely that the majority of the somatic hybrid mitogenomes originated from Sc (Fig. 2).

**Figure 3:**
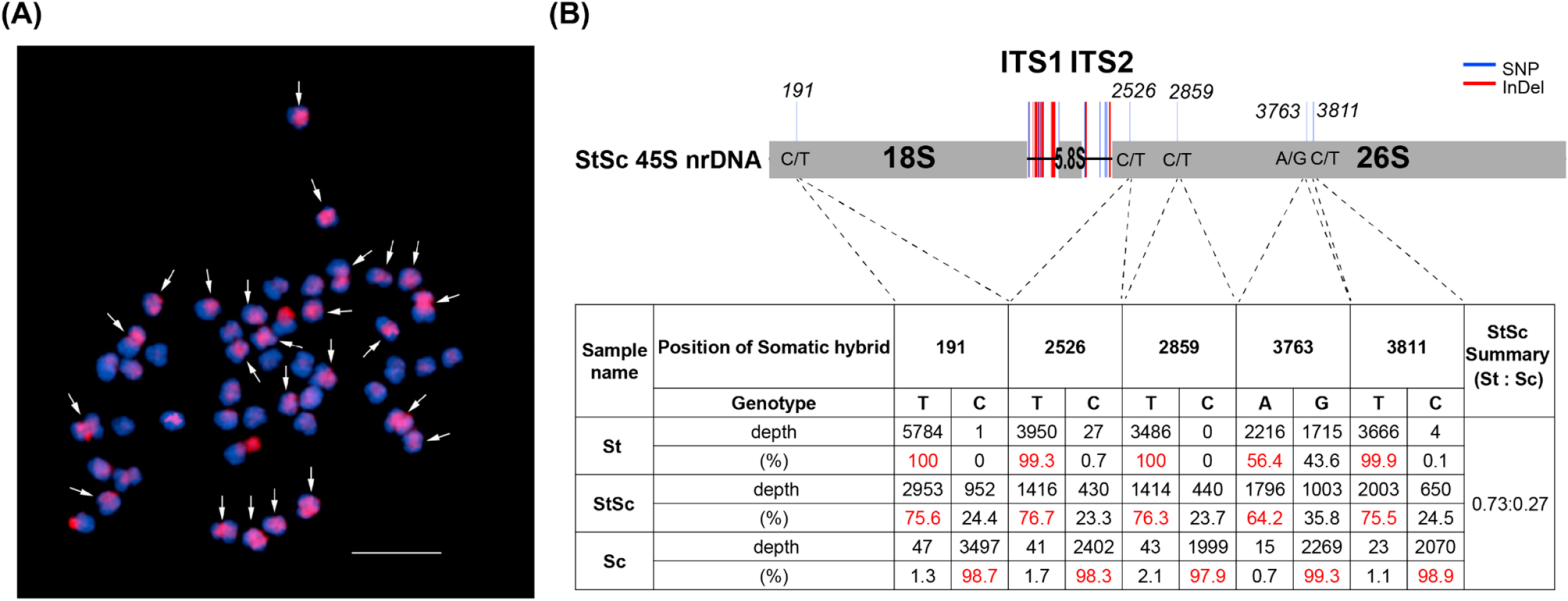
Detection of nuclear genome fusion in somatic hybrid. (**A**) GISH analysis of somatic hybrid (HA06-1 clone) using *S. tuberosum* specific-probes. The red signal of 24 arrows indicates the *S. commersonii* nuclear subgenomic distribution. (**B**) Schematic diagram of 45S ribosomal DNA cistron of *Solanum* species. StSc summary represents the percentage of St or Sc genotypes in the 45SnrDNA sequence.

GISH data using Sc genome probes revealed strong signals in 24 chromosomes but weak signals in the other 24 chromosomes in the StSc somatic hybrid (Fig. 3A). We also assembled and compared 45S nrDNA cistron sequence of three species. For example, multiple aligned position at 191 bp represents ‘T’ genotype in St and ‘C’ genotype in Sc. However, in StSc, it was identified that 75.6% of ‘T’ and 24.4% of ‘C’ were present. In conclusion, the overall 45S nrDNA sequences of StSc revealed both genotypes with average about 70 and 30 ratio for Sc and St, respectively (Fig. 3B).

**Figure 4:**
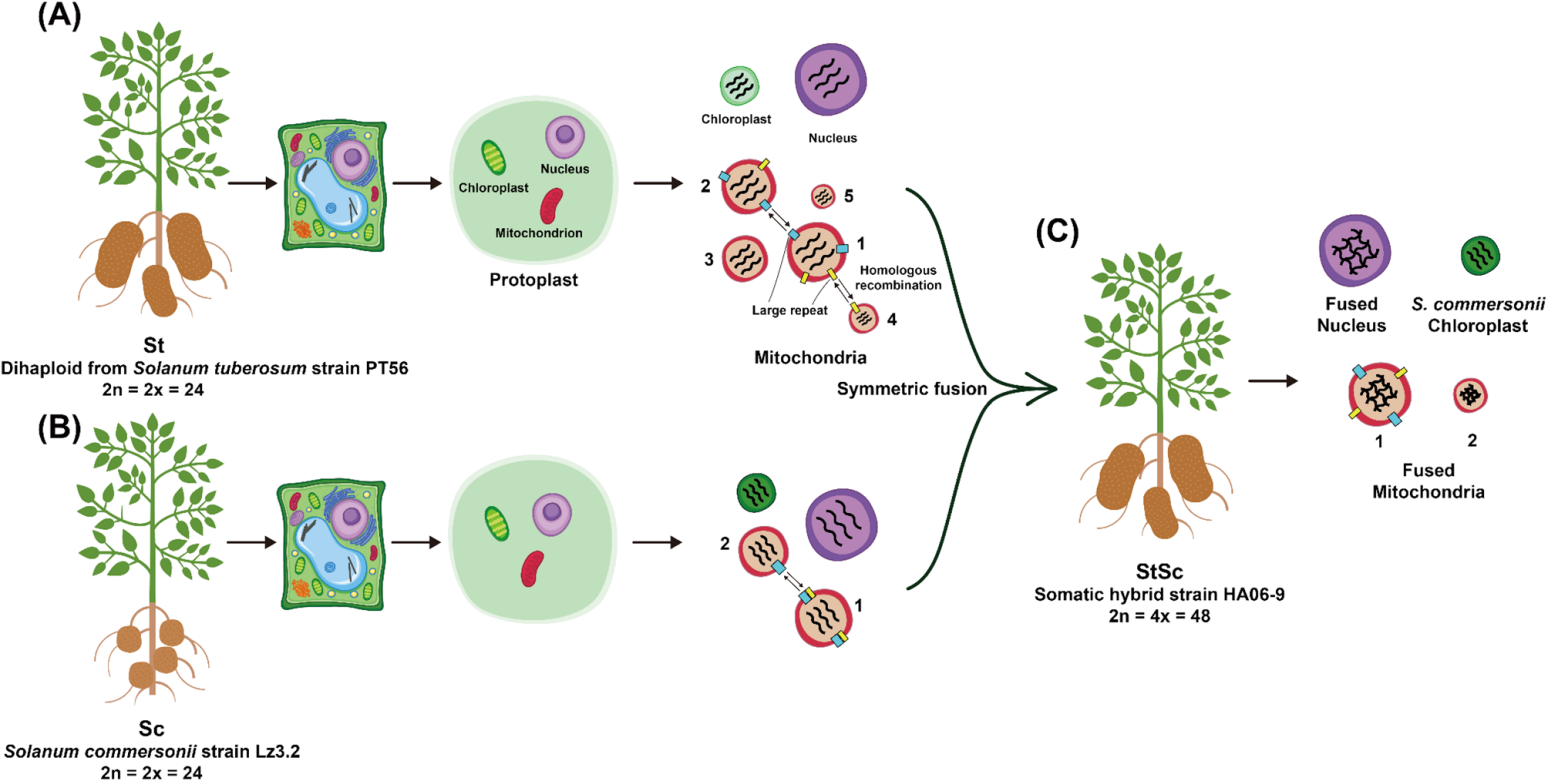
Schematic diagram of mitogenome in parental species and their somatic hybrids. (**A**) *S. tuberosum* (St), (**B**) *S. commersonii* (Sc)*,* and (**C**) somatic hybrid (StSc). *S. tuberosum* and *S. commersonii* have five and two subgenomes, respectively, which are fused into two subgenomes in the somatic hybrid generated by protoplast fusion. The origin of chloroplast genome in somatic hybrid has been determined based on sequence comparison among chloroplast genome sequences of parental species and that of the somatic hybrid.

In summary, St, a dihaploid of tetraploid cultivated potato, has five mitogenomes. Sc, a diploid wild potato, has two mitogenomes. Somatic hybrids developed via protoplast fusion of these two diploids contain the Sc-unique plastome^37^ but recombined mitogenomes and nuclear genomes derived from both St and Sc genomes (Fig. 4).

### Mutation rate of mitochondrial genes in Solanaceae

**Figure 5:**
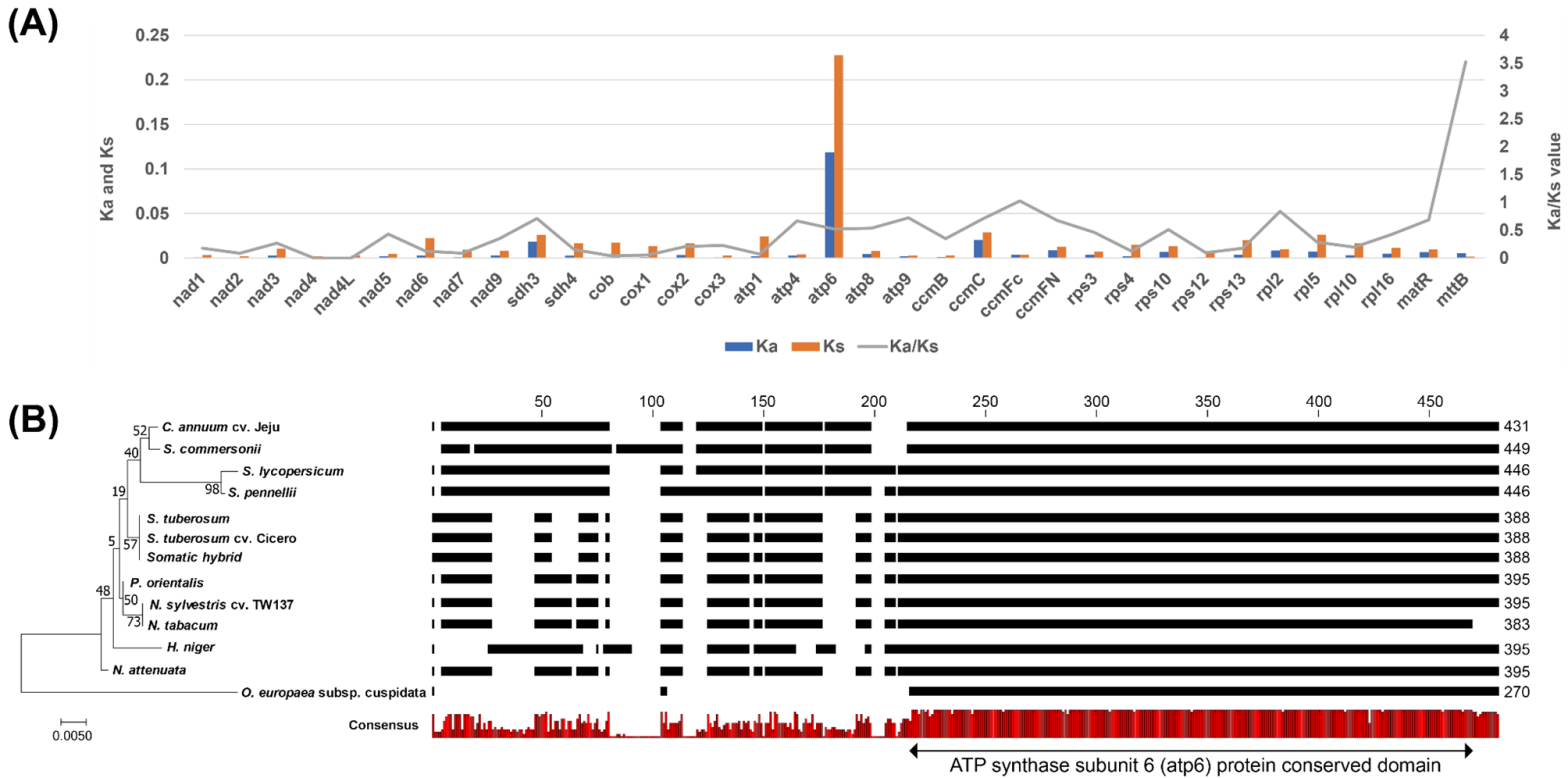
Mitochondrial gene diversity in Solanaceae family. (**A**) non-synonymous substitution (Ka) and synonymous substitution (Ks) values among the 12 Solanaceae species. Ka and Ks values were calculated with 35 protein-coding genes by CodeML program. (**B**) Variations of *atp6* are shown by the phylogenetic tree and multiple comparisons of amino acid sequences. The conserved domain has been determined through NCBI BLASTP search.

A total of 35 PCGs were common across Solanaceae. The nonsynonymous substitution (Ka), synonymous substitution (Ks), and their ratios were calculated. The Ka values ranged from 0 to 0.119 with a 0.003 of median value. The *nad4* and *nad4L* genes had the lowest Ka values, while *atp6* had the highest Ka value. The Ks values ranged from 0.02 to 0.228 with a 0.01 of median value. Moreover, *mttB* and *atp6* had the lowest and highest Ks values, respectively. Lastly, the Ka/Ks values ranged from 0 to 3.528 with a median value of 0.286 (Table S8, Fig. 5A). A Ka/Ks value of more than 2 was observed due to the extremely low Ks value.

Although the Ka and Ks values were generally low, *ccmFc* and *mttB* exhibited high Ka/Ks values of more than 1, indicating that these genes were positively selected during evolution (Fig. 5A). Considering that *atp6* showed a high mutation rate above 0.1. Ka and Ks values relative to the other genes, the amino-acid sequences corresponding to *atp6* were compared among Solanaceae species, which revealed that amino acid sequences were variable at the N-terminus but conserved at the C-terminus (Fig. 5B).

**Figure 6:**
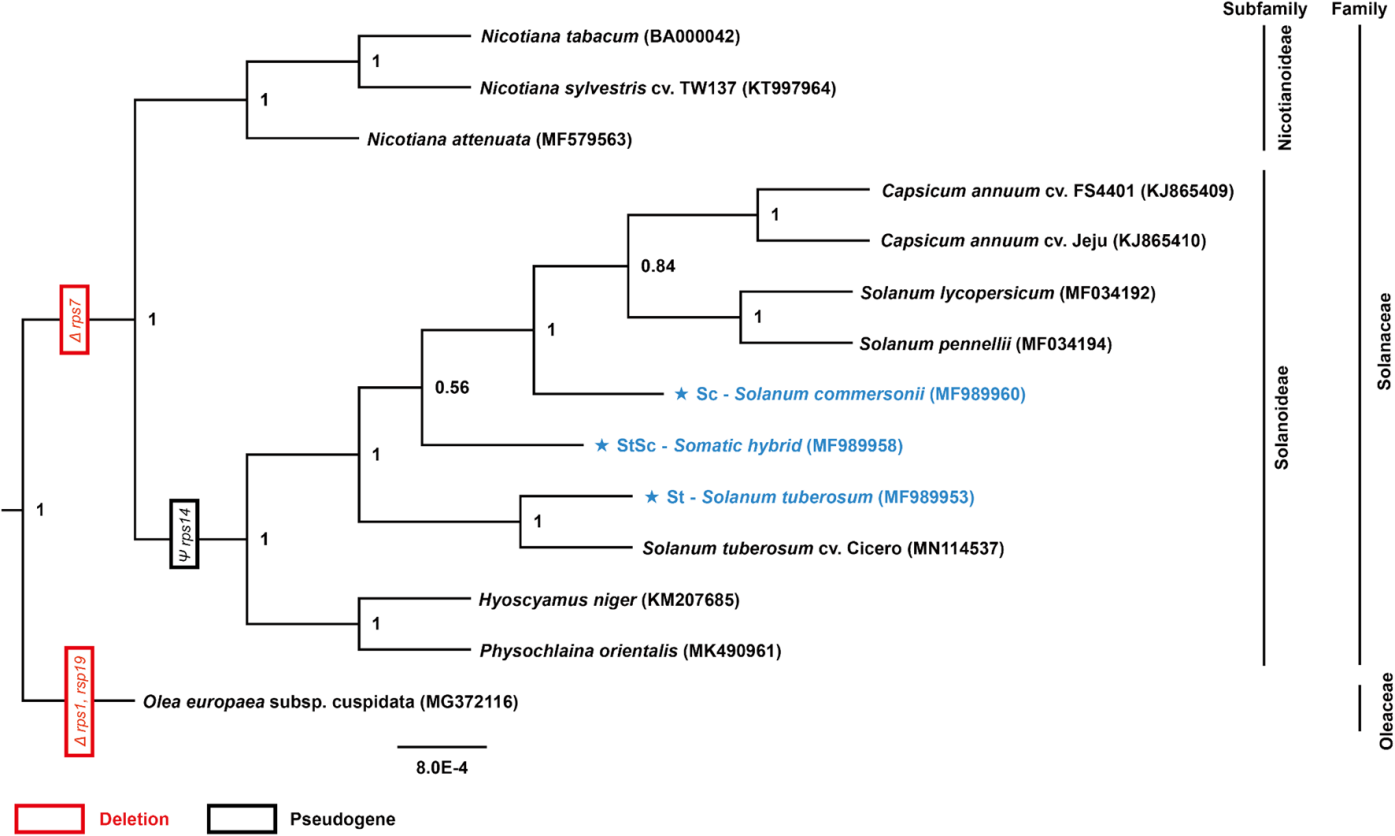
Phylogenetic relationship of 13 Solanaceae species using 35 protein-coding gene sequences commonly conserved in mitogenomes. The maximum likelihood tree was constructed using RAxML program with GTR + Γ + I model (based on jModelTest2) and a bootstrapping value of 1000. The bootstrap value (> = 0.5) is shown on the node. Deleted genes and pseudogenes specifically within each group in the tree have been also shown by red and black boxes, respectively. *Olea europaea* in the Oleaceae family has been used as an out-group.

Phylogenetic trees were constructed using various programs, including RAxML, MEGA7, PhyML, and BEAST to examine the topology of the species. Trees treated with RAxML, PhyML, and BEAST displayed the same topology, while those treated with MEGA7 exhibited slightly different topologies (Fig. S3). In trees generated using RAxML representing an optimized topology (Figs. 6 and S3), Solanaceae species were divided into two subfamilies, Solanoideae and Nicotianoideae, and the somatic hybrid exhibited a moderate branch between St and Sc. During the evolution of Solanaceae mitogenome, first, *rps1* and *rps19* were present in Solanaceae, however, these were omitted completely in Oleaceae. Next, *rps7* was confirmed to be completely deleted in Solanaceae compared to Oleaceae. Lastly, *ycf14* in all Nicotianoideae species was pseudogenized in the divergence period between Solanoideae and Nicotianoideae (Fig. 6).

## Discussion

### Diverse mitogenome structures in potato

The potato (*S. tuberosum*) nuclear genome was reported by the Potato Genome Sequencing Consortium^40^, and mitogenomes were reported by our group for *S. tuberosum* (MF989953-MF989957) and *S. commersonii* (MF989960-MF989961)^20^. In this study, we sequenced a novel mitogenome for somatic hybrids (MF989958-MF989959) of St and Sc and conducted a comparative genome analysis. These assemblies play an important role in the unique genetic inheritance patterns of somatic hybrid mitogenomes. Plant mitogenomes exhibit dynamic recombinant structures mediated by HR^41,42^. The large repeats shared in St, Sc, and StSc mitogenomes might facilitate HR events in St, Sc, and StSc.

The HR of mitogenomes mediated by dispersed repeats was suggested to consist of a multivariate configuration and subgenomes^43^. Similar to the five sub-mitogenomes of *S. tuberosum* (accession no. PT56: St), another mitogenome was reported using the cultivar Cicero and Désirée (hereafter referred to as Cicero) based on PacBio sequencing^44^. Meanwhile, our assembled and Cicero mitogenomes exhibited noticeable differences. The largest mitogenome of Cicero is the fused form of mitogenome subgenomes 1, 2, and 4 (Fig. S5). Our mitogenome subgenome-1 was assembled in a circular form; in contrast, the Cicero’s largest mitogenome was observed in the linear form. The St sub-mitogenomes 1, 2, and 4 shared large repeats that could mediate HR (Figs. 1 and 4), suggesting that various sub-mitogenomes can exist in different individuals or tissues.

Furthermore, *atp6* in St sub-mitogenome 1 was prematurely terminated as 321 amino acids (aa) in length, whereas the Cicero mitogenome had a full structure gene of 389 aa. The discrepancy of *atp6* was examined thoroughly, and a linear fragment with a complete structure of 389 aa was found by mapping to *atp6* sequence of Cicero mitogenome.

Not only master circle, but also other forms due to homologous recombination of mitochondrial subgenomes exist in the different cells^45^. This suggests that there might be a different form or an unrevealed linear form or short fragment could also contain essential genes in the mitogenome, such as *atp6*, even if not in the circular form.

### Transmission of plastome and mitogenomes in somatic hybrid

The chloroplasts of somatic hybrids were randomly selected and delivered from parents, and the nuclei or mitochondria are known to fuse^13^. In previous study, the StSc plastome was transferred only from *S. commersonii*^37^. In addition, StSc mitogenomes were randomly rearranged between St and Sc in most regions. The clustering analysis of CDSs revealed that the majority of the genes were derived from Sc; however, certain genes were derived from St (Fig. 2).

Previous studies have suggested that the somatic hybrids harbor recombinant mitogenomes that share both mitogenome types based on fingerprinting patterns^13,24,32,33^. In this study, we have displayed sequence-level recombination events that share unique genes from each of the parental species of somatic hybrids. Although this phenomenon may have occurred entirely randomly, fundamental mitochondrial genes in the Solanaceae family were preserved. Although the recombination mechanism is unclear, we assume that the smaller mitogenome might be competitive during somatic hybridization.

### Low evolutionary rate of mitochondrial genes in Solanaceae

To date, missing or misnamed genes have been examined in the Solanaceae mitogenome, and it has been confirmed that 35 PCGs are commonly preserved (Table S7). These genes were found to have few mutations, and even if mutations existed, most of them were identified as synonymous substitutions. This result is consistent with the fact that mitogenomes have few gene variations^38,46^. Exceptionally, *atp6* exhibited large length variation. Moreover, *atp6* was present in an intact form with a short length; however, the Solanaceae mitogenome evolutionary process confirmed that the sequence continued to accumulate in the front of the conserved motif (Fig. 5). It was considered that *atp6* could continuously promote mutations, suggesting that it can be useful for Solanaceae DNA barcoding. Novel ORFs that might be created during somatic hybridization have been identified; however, their function is unknown.

Based on the NCBI land plant organelle database (https://www.ncbi.nlm.nih.gov/genome/organelle/), 5295 cp genomes have been published, whereas only 279 mitogenomes have been released (July 2021). This can be attributed to the difficulty in assembling the mitogenome compared to the plastome. Therefore, our phylogenetic study will play an important role in identifying the relationship during mitochondrial evolution. Our phylogenetic trees were slightly different from those of traditional plastome-based trees^20^. The *Solanum*, *Capsicum*, *and Nicotiana* genera were assigned to the same lineage. However, in all other phylogenetic trees, *Capsicum* was grouped with *S. lycopersicum* and *S. pennellii*. This was due to the low mutation rate of the mitogenome.

## Materials and methods

### Plant materials and whole genome sequencing

The study complies with local and national guidelines. Plants of *Solanum tuberosum* (accession no. PT56: St), *S. commersonii* (accession no. Lz3.2: Sc), and their somatic hybrids (accession no. HA06-9: StSc) generated by protoplast fusion^47^ were used for the complete assembly of the mitogenome. All plants were grown and maintained at the Highland Agriculture Research Institute (HARI), RDA, Korea. H.-J. P., J.-H. S., J.-H. C., Y.-E. P., J.-G. C., and K.-S. C. prepared voucher specimen and identification. The voucher specimen of Sc, St and StSc was assigned HLP1841, HLP1842, and HLP1843, respectively in Potato Germplasm Center, Korea.

The total genomic DNA was extracted from fresh leaves using DNeasy Plant MiniKit (Qiagen, CA, USA) according to the manufacturer’s instructions and examined using NanoDrop (DeNovix, Wilmington, USA) and 2100 Expert Bioanalyzer (Agilent Technologies, USA). Paired-end (PE) genomic libraries were constructed according to the standard Illumina PE protocol and sequenced using an Illumina MiSeq platform (Illumina, San Diego, CA) by Macrogen Biotechnology Center (Marcrogen Inc., Seoul, Korea, http://www.macrogen.com/).

### De novo assembly and validation of mitochondrial genomes and 45S nuclear ribosomal DNA

Raw PE data of approximately 5.8 Gb for *S. tuberosum*, 6.6 Gb for *S. commersonii*, and 5.3 Gb for somatic hybrid were generated and used for assembly (Table S1). De novo mitogenome assembly was performed using PE data according to a previous study^48^. Briefly, high-quality read sequences (Phred score > 20) were obtained, and de novo assembly was conducted using the clc_novo_assemble tool in the CLC Assembly Cell package (ver. 4.2.1, CLC Inc., Denmark). Contigs derived from raw data of mitogenomes were selected based on similarity with mitogenome sequences of other Solanaceae species, such as *Capsicum annuum* (GenBank acc. no. KJ865409), and *Nicotiana tabacum* (GenBank accession No. no. KR780036), and then extended, gap-filled, and merged through a series of read mapping to generate a draft circular mitogenome sequence.

The draft mitogenome sequences were validated using bioinformatics and experimental methods. For validation based on PE read mapping, the high-quality PE reads were mapped again on the draft mitogenome sequences; subsequently, the consistency and connectivity of the mapped reads on draft mitogenomes and on junctions between contigs were confirmed. In read mapping, the coexistence of chloroplast or nuclear genome-derived reads was manually confirmed and removed based on extremely high or low depth.

For validation based on PCR amplification and nucleotide sequencing, specific primers for each subgenome were designed using the NCBI Primer-BLAST tool (https://www.ncbi.nlm.nih.gov/tools/primer-blast/index.cgi) and used for genomic DNA PCR amplification and nucleotide sequencing (Table S2). For PCR analysis, 10 ng of genomic DNA was used in a 20-µL PCR mixture of AccuPower PCR PreMix (Bioneer, Daejeon, Korea) that consists of 0.2 U/µL TOP DNA polymerase, 1.5 mM Mg^2+^, and 250 μM each dNTP mixture with 5 pmol of each primer. PCR conditions were as follows: 95 °C for 5 min; 25 cycles of 95 °C for 30 s, 58 °C for 30 s, and 72 °C for 1 min; and 72 °C for 10 min. PCR products were analyzed by 1.8% agarose gel electrophoresis. The nucleotide sequences of the PCR amplicons were determined by Sanger sequencing and compared with the draft mitogenome sequence for validation.

### Mitochondrial genome annotation

Mitogenomes were initially annotated using the GeSeq program (https://chlorobox.mpimp-golm.mpg.de/geseq-app.html)^49^, and the genes were further predicted by comparison with mitogenomes of other Solanaceae species such as *Capsicum annuum* (GenBank acc. no. KJ865409) and *Nicotiana tabacum* (GenBank accession No. no. KR780036). Ambiguous gene positions were manually corrected using NCBI BLASTN-based search analysis and the Artemis annotation tool^50^. A linear and circular map of the mitogenome with annotation information was drawn using the OGDRAW program (https://chlorobox.mpimp-golm.mpg.de/OGDraw.html)^51^.

Repetitive sequences such as direct and palindrome repeats in the mitogenome were searched using the Vmatch program (http://www.vmatch.de)^52^ integrated with the REPuter program with a minimum repeat length of 20 bp and then selected using a length cut-off value of 100 bp. Tandem repeats were searched using the Tandem Repeats Finder program with parameters such as match 2, mismatch 7, indels 7, minimum alignment score 80, maximum period size 10, and maximum TR size 50 (https://tandem.bu.edu/trf/trf.html)^53^.

### Identification of genome collinearity and sequence variation between mitochondrial genomes

Genome collinearity regions among mitogenomes were identified by reciprocal BLASTN searches with a cutoff e-value of 1e-1 and a minimum length of 1000 bp (Table S3). In addition, the origin of somatic hybrid mitogenome sequences was inferred by comparison with *S. tuberosum* and *S. commersonii* mitogenome sequences using Mega BLASTN searches with parameters of cut-off e-value 1e-1 and minimum match length 1000 bp. A collinearity map was visualized using a Circos program in R package and OGDRAW program (https://chlorobox.mpimp-golm.mpg.de/OGDraw.html)^51^. Mitogenome sequences possibly derived from chloroplast and nuclear genomes were identified using BLASTN-based analysis with a cutoff e-value of 1E-1 against *S. tuberosum* chloroplast (GenBank accession no. JF772171) and nuclear genomes (SolTub_3.0, https://www.ncbi.nlm.nih.gov/assembly/GCF_000226075.1/).

### Phylogenetic analysis and calculation of nucleotide substitution rate

The phylogenetic analysis based on the maximum likelihood (ML) was performed using conserved 35 protein-coding gene (PCG) sequences (*nad1, nad2, nad3, nad4, nad4L, nad5, nad6, nad7, nad9, sdh3, sdh4, cob, cox1, cox2, cox3, atp1, atp4, atp6, atp8, atp9, ccmB, ccmC, ccmFc, ccmFN, rps3, rps4, rps10, rps12, rps13, rpl2, rpl5, rpl10, rpl16, matR,* and *mttB*) in 14 species (13 in Solanaceae and one out group in Oleaceae) belonging to the Asterids group (Table S4). Concatenated PCG sequences were aligned using the MAFFT program (ver. 7)^54^. To find the best substitution models, the jModelTest program-based (ver. 2.1.10) analysis was conducted using the Akaike information criterion (AIC), Bayesian information criterion (BIC), and invariable site options^55^. ML phylogenetic trees were constructed, and various programs were compared, such as, RAxML (ver. 8.2.12)^56^, MEGA (ver. 7)^57^, and PhyML (ver. 3)^58^ programs. A Bayesian phylogenetic tree was constructed using the BEAST (ver. 2.6.2)^59^ and MrBayes (ver. 3.2.7)^60^ programs. Finally, the selected phylogenetic analysis was conducted using RAxML (ver. 8.2.12) with the GTR + Γ + I (invariable) nucleotide substitution model, 1000 bootstraps, and 1000 random number seed options^56^. A phylogenetic tree was constructed using the FigTree program (ver. 1.4.2, http://tree.bio.ed.ac.uk/software/figtree/). The non-synonymous substitution (Ka) and synonymous substitution (Ks) ratios were calculated for the 35 PCG sequences using the CodeML tool in the PAML software package^61^.

### Genomic in situ hybridization (GISH)

*S. tuberosum* (accession no. PT56) gDNA (1 µg) was labeled with Texas Red-5-dUTP (Perkin Elmer, NEL417001EA) and Alexa Fluor 488-5-dUTP (Invitrogen, C11397), through direct nick translation. These labeled gDNAs were used as probes for GISH. Slides with metaphase chromosomes of somatic hybrids were fixed with 4% paraformaldehyde for 5 min, dehydrated in an ethanol series (70–100%), and air-dried. The probe hybridization mixture contained 50% formamide, 10% dextran sulfate, 2 × SSC, 20 ng of each gDNA probe, and DNase-free water. Probes were denatured at 90 °C for 10 min and immediately placed on ice for at least 5 min prior to mounting on slides with good metaphase chromosome spreads. A total volume of 40μL was mounted per slide. Next, chromosomes were co-denatured with the probes at 80 °C for 3–5 min on a ThermoBrite (Fisher Scientific, USA) and hybridized overnight at 37 °C. Slides were then washed in 2 × SSC for 5 min at RT, 0.1 × SCC for 25 min at 42 °C, and 2 × SSC for 5 min at 20 °C. After ethanol series (70%, 90%, 100%) dehydration for 2 min each, the slides were air-dried and counterstained with 1 μg/mL DAPI in Vectashield (Vector Laboratories, USA). Images were captured using an Olympus BX53 fluorescence microscope equipped with a Leica DFC365 FS CCD camera and processed using Cytovision (ver. 7.2, Leica Microsystems, Germany). We performed further image enhancements using Adobe Photoshop CC.

## Supplementary Information

Supplementary Information.

## Data Availability

The complete mitogenome have been deposited in the National Center for Biotechnology Information (NCBI) database under GenBank accession numbers MF989958-MF989959 for Somatic Hybrid, MF989960-MF989961 for *S. commersonii*, and MF989953-MF989957 for *S. tuberosum*.
