# Supplementary material for: Mitochondrial genome recombination in somatic hybrids of Solanum commersonii and S. tuberosum
Source: Sci Rep. 2022 May 23;12:8659. doi: 10.1038/s41598-022-12661-z (PMC9127095; doi:10.1038/s41598-022-12661-z)
Supplement: Supplementary file 1 — Supplementary Information. [file 41598_2022_12661_MOESM1_ESM.docx]

# **Supplementary Tables**

**Table S1.** Summary of Illumina sequencing data for the *de novo* assembly of mitogenome of parental species, *S. tuberosum* and *S. commersonii.* and their somatic hybrid

| Category | Somatic Hybrid | | *S. tuberosum* | | | | | *S. commersonii* | |
| --- | --- | --- | --- | --- | --- | --- | --- | --- | --- |
| Total raw read numbers | 17,750,412 | | 19,368,194 | | | | | 22,056,066 | |
| Total raw bases | 5,342,874,012 | | 5,807,568,326 | | | | | 6,597,040,721 | |
| Total trimmed read numbers | 15,483,215 | | 16,424,842 | | | | | 19,292,972 | |
| Total trimmed bases | 3,602,925,285 | | 4,065,781,506 | | | | | 4,831,428,596 | |
| Mitogenome types | 1 | 2 | 1 | 2 | 3 | 4 | 5 | 1 | 2 |
| Mapped read numbers on Mitogenome | 152,191 | 23,070 | 41,344 | 36,484 | 31,161 | 5,411 | 12,729 | 130,811 | 80,817 |
| Average coverage (x) | 81.73 | 98.48 | 33.07 | 34.99 | 65.58 | 26.16 | 61.47 | 90.87 | 88.27 |

**Table S2**. The list of primers used for validation of the mitogenomes obtained by *de novo* assembly.

| Species | Primers No. | Direction | Sequences (5' to 3') | Expected amplicon size (bp) | Positions | Locations | Mt type |
| --- | --- | --- | --- | --- | --- | --- | --- |
| *S. tuberosum* | st_1_1 | Forward | TTTGTTGTGCGAAGAGTGCG | 840 | nad5 intron | 31443-31462 | I |
|  |  | Reverse | GTGCTCTAGCAATTGCGCTC |  |  | 32263-32282 |  |
|  | st_1_2 | Forward | CGAAAAGCGAGATGAGCGTG | 1,004 | nad4 intron | 87446-87465 |  |
|  |  | Reverse | CAGAAATCCCGCATTGAGCG |  |  | 88430-88449 |  |
|  | st_1_3 | Forward | GAGCGGGAATACGTCGCTTA | 375 | atp1~orf101 | 144881-144900 |  |
|  |  | Reverse | GTTCCGTGGGTTCAGTTTGC |  |  | 145236-145255 |  |
|  | st_1_4 | Forward | CATAAGCGTGAAAGAGCCGC | 747 | nad7~trnM-CAU | 190188-190207 |  |
|  |  | Reverse | GGACGAGACGCTAACCTACG |  |  | 190915-190934 |  |
|  | st_1_5 | Forward | CGATCGGGCCTTACTCACTC | 805 | nad1~orf125 | 228542-228561 |  |
|  |  | Reverse | CATTCTTTCGCGTGCCGTAG |  |  | 229327-229346 |  |
|  | st_1_6 | Forward | AGTCCAGGAGGCTAGTCTGG | 1,263 | trnM-CAU~trnL-CAA | 271567-271586 |  |
|  |  | Reverse | CTCGAATGCGCTTACCTTGC |  |  | 272810-272829 |  |
|  | st_3_1 | Forward | CGCTTACGAAATGAAGCCCG | 472 | orfB~trnV-GAC | 9946-9965 | III |
|  |  | Reverse | GGGGTCTCCCCCTATCAACT |  |  | 10398-10417 |  |
|  | st_3_2 | Forward | GCGATCAAGTCGTTCGCTTC | 738 | orf140~ccmFc | 63636-63655 |  |
|  |  | Reverse | TGCGTTTCACGCTTTTCAGG |  |  | 64354-64373 |  |
|  | st_3_3 | Forward | GGAATCCTTGCTCCTGGCTT | 822 | trnS-UGA~rRNA | 104592-104611 |  |
|  |  | Reverse | CGTCGATCGAACGAAAACCG |  |  | 105394-105413 |  |
|  | st_5_1 | Forward | ATGGAGTGGGGAATAGCCCT | 364 | nad2 | 30449-30468 | V |
|  |  | Reverse | CGGATTTGCGTGAACTAGCG |  |  | 30793-30812 |  |
| *S. commersonii* | sc_1_1 | Forward | CATTCTTTCGCGTGCCGTAG | 805 | orf125~nad1 | 41432-41451 | I |
|  |  | Reverse | CGATCGGGCCTTACTCACTC |  |  | 42217-42236 |  |
|  | sc_1_2 | Forward | CTAGCGCTTCGCGTTCTTTC | 1,414 | ccmFc intron | 76622-76641 |  |
|  |  | Reverse | TTCCCACAGGTGCCCTTTTT |  |  | 78016-78035 |  |
|  | sc_1_3 | Forward | GGAATCCTTGCTCCTGGCTT | 822 | trnS-UGA~orf123 | 107505-107524 |  |
|  |  | Reverse | CGTCGATCGAACGAAAACCG |  |  | 108307-108326 |  |
|  | sc_1_4 | Forward | TTTGTTGTGCGAAGAGTGCG | 840 | nad5 | 290814-290833 |  |
|  |  | Reverse | GTGCTCTAGCAATTGCGCTC |  |  | 291634-291653 |  |
|  | sc_2_1 | Forward | TGATTGGTTCCAGCCATCCC | 1,185 | trnN-GUU~trnC-GCA | 42577-42596 | II |
|  |  | Reverse | CGGCTATCCGAAAAACGCAG |  |  | 43742-43761 |  |
|  | sc_2_2 | Forward | CAGAAATCCCGCATTGAGCG | 1,004 | nad4 intron | 54393-54412 |  |
|  |  | Reverse | CGAAAAGCGAGATGAGCGTG |  |  | 55377-55396 |  |
| Somatic hybrid | sh_1_1 | Forward | CAGAAATCCCGCATTGAGCG | 1,004 | nad4 intron | 1436-1455 | I |
|  |  | Reverse | CGAAAAGCGAGATGAGCGTG |  |  | 2420-2439 |  |
|  | sh_1_2 | Forward | GTGCTCTAGCAATTGCGCTC | 840 | nad5 | 53335-53354 |  |
|  |  | Reverse | TTTGTTGTGCGAAGAGTGCG |  |  | 54155-54174 |  |
|  | sh_1_4 | Forward | CGATCGGGCCTTACTCACTC | 805 | nad1 intron | 174594-174613 |  |
|  |  | Reverse | CATTCTTTCGCGTGCCGTAG |  |  | 175379-175398 |  |
|  | sh_1_6 | Forward | GGACGAGACGCTAACCTACG | 747 | nad1 intron | 297263-297282 |  |
|  |  | Reverse | CATAAGCGTGAAAGAGCCGC |  |  | 297990-298009 |  |
|  | sh_1_7 | Forward | CTAAGGCACGCTCGATAGCA | 518 | nad1 intron | 312815-312834 |  |
|  |  | Reverse | GTGCGAAGCAAGTCTATGCG |  |  | 313313-313332 |  |
|  | sh_1_8 | Forward | TGATTGGTTCCAGCCATCCC | 1,185 | trnN-GUU~trnC-GCA | 388059-388078 |  |
|  |  | Reverse | CGGCTATCCGAAAAACGCAG |  |  | 389224-389243 |  |
|  | sh_2_1 | Forward | ATGGAGTGGGGAATAGCCCT | 364 | nad2 intron | 30432-30451 | II |
|  |  | Reverse | CGGATTTGCGTGAACTAGCG |  |  | 30776-30795 |  |

**Table S3**. Mitogenome homology analysis between mitogenomes of somatic hybrid and those of *S. tuberosum* and *S. commersonii*. Homologous sequences (synteny regions) among mitogenomes were identified by reciprocal BLASTN searches with cutoff e-value of 1E-1 and minimum length of 1,000 bp.

| Synteny 1 | Synteny 2 | Position of Synteny 1 | Position of Synteny 2 | Match length (bp) (>1kb) | % identity | Match strand |
| --- | --- | --- | --- | --- | --- | --- |
| Somatic hybrid mt type1 | S. commersonii mt type1 | 88105-216829 | 128725-1 | 128725 | 100.00 | - |
| Somatic hybrid mt type1 | S. commersonii mt type1 | 278990-344663 | 200840-135167 | 65674 | 100.00 | - |
| Somatic hybrid mt type1 | S. commersonii mt type1 | 34476-78250 | 310512-266738 | 43775 | 99.99 | - |
| Somatic hybrid mt type1 | S. commersonii mt type1 | 216830-235476 | 338427-319782 | 18647 | 99.98 | - |
| Somatic hybrid mt type1 | S. commersonii mt type1 | 327804-345853 | 298594-316643 | 18050 | 99.98 | + |
| Somatic hybrid mt type1 | S. commersonii mt type1 | 262324-279325 | 266740-249741 | 17002 | 99.94 | - |
| Somatic hybrid mt type1 | S. commersonii mt type1 | 34476-46391 | 140108-152023 | 11916 | 100.00 | + |
| Somatic hybrid mt type1 | S. commersonii mt type1 | 249405-261273 | 72589-84456 | 11869 | 99.93 | + |
| Somatic hybrid mt type1 | S. commersonii mt type1 | 228699-235476 | 135310-128535 | 6778 | 99.94 | - |
| Somatic hybrid mt type1 | S. commersonii mt type1 | 88105-91710 | 319972-316373 | 3606 | 99.75 | - |
| Somatic hybrid mt type1 | S. commersonii mt type1 | 287198-288259 | 50861-49800 | 1062 | 99.62 | - |
| Somatic hybrid mt type1 | S. commersonii mt type1 | 165969-167030 | 192632-191571 | 1062 | 99.62 | - |
| Somatic hybrid mt type1 | S. commersonii mt type2 | 278990-339722 | 148164-87432 | 60733 | 100.00 | - |
| Somatic hybrid mt type1 | S. commersonii mt type2 | 345483-398439 | 1-52957 | 52957 | 99.99 | + |
| Somatic hybrid mt type1 | S. commersonii mt type2 | 1-46391 | 52958-99347 | 46391 | 100.00 | + |
| Somatic hybrid mt type1 | S. commersonii mt type2 | 262712-279325 | 213676-197065 | 16614 | 99.93 | - |
| Somatic hybrid mt type1 | S. commersonii mt type2 | 261124-262711 | 1588-1 | 1588 | 100.00 | - |
| Somatic hybrid mt type1 | S. commersonii mt type2 | 165969-167030 | 139956-138895 | 1062 | 99.62 | - |
| Somatic hybrid mt type1 | S. tuberosum mt type1 | 5366-64359 | 80231-21281 | 59002 | 99.81 | - |
| Somatic hybrid mt type1 | S. tuberosum mt type1 | 167350-224380 | 221298-278325 | 57041 | 99.90 | + |
| Somatic hybrid mt type1 | S. tuberosum mt type1 | 345428-398439 | 142902-89884 | 53022 | 99.93 | - |
| Somatic hybrid mt type1 | S. tuberosum mt type1 | 291040-343586 | 197157-144614 | 52549 | 99.92 | - |
| Somatic hybrid mt type1 | S. tuberosum mt type1 | 271938-288256 | 204731-221041 | 16319 | 99.93 | + |
| Somatic hybrid mt type1 | S. tuberosum mt type1 | 327804-339722 | 39214-51132 | 11919 | 99.95 | + |
| Somatic hybrid mt type1 | S. tuberosum mt type1 | 34476-46391 | 148478-160393 | 11916 | 99.96 | + |
| Somatic hybrid mt type1 | S. tuberosum mt type1 | 186393-193895 | 289511-297014 | 7504 | 99.96 | + |
| Somatic hybrid mt type1 | S. tuberosum mt type1 | 144035-150653 | 6011-12629 | 6619 | 99.96 | + |
| Somatic hybrid mt type1 | S. tuberosum mt type1 | 1-5368 | 89883-84517 | 5368 | 99.96 | - |
| Somatic hybrid mt type1 | S. tuberosum mt type1 | 225165-229915 | 278310-283048 | 4754 | 99.01 | + |
| Somatic hybrid mt type1 | S. tuberosum mt type1 | 153212-157219 | 197726-201749 | 4032 | 99.03 | + |
| Somatic hybrid mt type1 | S. tuberosum mt type1 | 158407-161348 | 15557-12622 | 2942 | 99.66 | - |
| Somatic hybrid mt type1 | S. tuberosum mt type1 | 261124-263926 | 141260-144057 | 2806 | 99.22 | + |
| Somatic hybrid mt type1 | S. tuberosum mt type1 | 230188-232569 | 283074-285465 | 2392 | 99.12 | + |
| Somatic hybrid mt type1 | S. tuberosum mt type1 | 165969-167320 | 219983-221334 | 1352 | 99.78 | + |
| Somatic hybrid mt type1 | S. tuberosum mt type1 | 36276-38463 | 2016-4205 | 2227 | 73.73 | + |
| Somatic hybrid mt type1 | S. tuberosum mt type1 | 335735-337922 | 4205-2016 | 2227 | 73.73 | - |
| Somatic hybrid mt type1 | S. tuberosum mt type1 | 55528-56767 | 2095-3336 | 1257 | 78.04 | + |
| Somatic hybrid mt type1 | S. tuberosum mt type2 | 5366-64359 | 80231-21281 | 59002 | 99.81 | - |
| Somatic hybrid mt type1 | S. tuberosum mt type2 | 345428-398439 | 142902-89884 | 53022 | 99.93 | - |
| Somatic hybrid mt type1 | S. tuberosum mt type2 | 291040-343586 | 197157-144614 | 52549 | 99.92 | - |
| Somatic hybrid mt type1 | S. tuberosum mt type2 | 167350-193895 | 221298-247843 | 26546 | 99.96 | + |
| Somatic hybrid mt type1 | S. tuberosum mt type2 | 271938-288256 | 204731-221041 | 16319 | 99.93 | + |
| Somatic hybrid mt type1 | S. tuberosum mt type2 | 327804-339722 | 39214-51132 | 11919 | 99.95 | + |
| Somatic hybrid mt type1 | S. tuberosum mt type2 | 34476-46391 | 148478-160393 | 11916 | 99.96 | + |
| Somatic hybrid mt type1 | S. tuberosum mt type2 | 144035-150653 | 6011-12629 | 6619 | 99.96 | + |
| Somatic hybrid mt type1 | S. tuberosum mt type2 | 1-5368 | 89883-84517 | 5368 | 99.96 | - |
| Somatic hybrid mt type1 | S. tuberosum mt type2 | 153212-157219 | 197726-201749 | 4032 | 99.03 | + |
| Somatic hybrid mt type1 | S. tuberosum mt type2 | 158407-161348 | 15557-12622 | 2942 | 99.66 | - |
| Somatic hybrid mt type1 | S. tuberosum mt type2 | 261124-263926 | 141260-144057 | 2806 | 99.22 | + |
| Somatic hybrid mt type1 | S. tuberosum mt type2 | 165969-167320 | 219983-221334 | 1352 | 99.78 | + |
| Somatic hybrid mt type1 | S. tuberosum mt type2 | 36276-38463 | 2016-4205 | 2227 | 73.73 | + |
| Somatic hybrid mt type1 | S. tuberosum mt type2 | 335735-337922 | 4205-2016 | 2227 | 73.73 | - |
| Somatic hybrid mt type1 | S. tuberosum mt type2 | 55528-56767 | 2095-3336 | 1257 | 78.04 | + |
| Somatic hybrid mt type1 | S. tuberosum mt type3 | 106905-144241 | 107012-69680 | 37338 | 99.91 | - |
| Somatic hybrid mt type1 | S. tuberosum mt type3 | 67860-97178 | 33383-4074 | 29320 | 99.88 | - |
| Somatic hybrid mt type1 | S. tuberosum mt type3 | 232568-261273 | 52834-81548 | 28718 | 99.85 | + |
| Somatic hybrid mt type1 | S. tuberosum mt type3 | 101191-106956 | 112800-107031 | 5770 | 99.74 | - |
| Somatic hybrid mt type1 | S. tuberosum mt type3 | 97114-101190 | 795136 | 4077 | 99.88 | - |
| Somatic hybrid mt type1 | S. tuberosum mt type3 | 65335-67467 | 37744-35612 | 2133 | 100.00 | - |
| Somatic hybrid mt type1 | S. tuberosum mt type3 | 111290-112878 | 35523-33935 | 1589 | 100.00 | - |
| Somatic hybrid mt type1 | S. tuberosum mt type4 | 193896-224380 | 1-30482 | 30495 | 99.84 | + |
| Somatic hybrid mt type1 | S. tuberosum mt type4 | 186393-193895 | 41668-49171 | 7504 | 99.96 | + |
| Somatic hybrid mt type1 | S. tuberosum mt type4 | 225165-229915 | 30467-35205 | 4754 | 99.01 | + |
| Somatic hybrid mt type1 | S. tuberosum mt type4 | 230188-232569 | 35231-37622 | 2392 | 99.12 | + |
| Somatic hybrid mt type2 | S. commersonii mt type1 | 4007-49206 | 200505-245734 | 45230 | 99.929 | + |
| Somatic hybrid mt type2 | S. commersonii mt type1 | 1-4342 | 245735-250076 | 4342 | 100 | + |
| Somatic hybrid mt type2 | S. commersonii mt type2 | 4007-49206 | 147829-193058 | 45230 | 99.929 | + |
| Somatic hybrid mt type2 | S. commersonii mt type2 | 1-4342 | 193059-197400 | 4342 | 100 | + |
| Somatic hybrid mt type2 | S. tuberosum mt type5 | 14370-49206 | 14384-49230 | 34862 | 99.639 | + |
| Somatic hybrid mt type2 | S. tuberosum mt type5 | 1-10427 | 1-10422 | 10427 | 99.866 | + |
| Somatic hybrid mt type2 | S. tuberosum mt type5 | 12536-14437 | 11736-13637 | 1902 | 99.895 | + |
| Somatic hybrid mt type2 | S. tuberosum mt type5 | 11161-12543 | 10329-11711 | 1383 | 99.783 | + |

**Table S4**. The GenBank accession numbers of mitogenomes used for comparative analysis

| No | Scientific name | GenBank accession nos. |
| --- | --- | --- |
| 1 | *Capsicum annuum* cultivar Jeju | KJ865410 |
| 2 | *Capsicum annuum* cultivar CMS line FS4401 | KJ865409 |
| 3 | *Hyoscyamus niger* | KM207685 |
| 4 | *Nicotiana attenuata* | MF579563 |
| 5 | *Nicotiana sylvestris* cultivar TW 137 | KT997964 |
| 6 | *Nicotiana tabacum* | BA000042 |
| 7 | *Olea europaea* subsp. cuspidata | MG372116 |
| 8 | *Physochlaina orientalis* | MK490961 |
| 9 | *Solanum commersonii* | MF989960, MF989961 |
| 10 | *Solanum lycopersicum* | MF034192 |
| 11 | *Solanum pennellii* | MF034194 |
| 12 | *Solanum tuberosum* | MF989953, MF989954, MF989955, MF989956,MF989957 |
| 13 | *Solanum tuberosum* cultivar Cicero | MN114537, MN114538, MN114539 |
| 14 | *Somatic hybrid* | MF989958, MF989959 |

**Table S5**. Distribution of direct and palindrome repeats (above 100 bp) of somatic hybrids and its parental species, *S. tuberosum* and *S. commersonii*. The repeat sequences were searched using Vmatch program with minimum repeat length of 20 bp (http://www.vmatch.de/) and selected by length cutoff value of 100 bp.

| Species | Mt type | No. | Repeat length (bp) | First region start | First region end | Second region start | Second region end | Direction |
| --- | --- | --- | --- | --- | --- | --- | --- | --- |
| *S. tuberosum* | I | 1 | 11916 | 39216 | 51132 | 148477 | 160393 | Palindromic |
|  |  | 2 | 7500 | 240343 | 247843 | 289514 | 297014 | Direct |
|  |  | 3 | 519 | 60859 | 61378 | 204194 | 204713 | Palindromic |
|  | III | 1 | 1589 | 33934 | 35523 | 101038 | 102627 | Direct |
|  |  | 2 | 146 | 7403 | 7549 | 55208 | 55354 | Direct |
|  |  | 3 | 124 | 21764 | 21888 | 67512 | 67636 | Palindromic |
| *S. commersonii* | I | 1 | 16857 | 135166 | 152023 | 298596 | 315453 | Palindromic |
|  |  | 2 | 10094 | 125171 | 135265 | 316418 | 326512 | Direct |
|  |  | 3 | 1024 | 49807 | 50831 | 191578 | 192602 | Direct |
|  |  | 4 | 432 | 206888 | 207320 | 277091 | 277523 | Direct |
|  |  | 5 | 407 | 266297 | 266704 | 316217 | 316624 | Direct |
|  |  | 6 | 336 | 200504 | 200840 | 249740 | 250076 | Direct |
|  |  | 7 | 206 | 125171 | 125377 | 266498 | 266704 | Direct |
|  |  | 8 | 156 | 27717 | 27873 | 263826 | 263982 | Palindromic |
|  |  | 9 | 146 | 122986 | 123132 | 320177 | 320323 | Palindromic |
|  |  | 10 | 146 | 122986 | 123132 | 128930 | 129076 | Palindromic |
|  |  | 11 | 140 | 59264 | 59404 | 280952 | 281092 | Direct |
|  |  | 12 | 135 | 59107 | 59242 | 280756 | 280891 | Direct |
|  |  | 13 | 128 | 49816 | 49944 | 124023 | 124151 | Palindromic |
|  |  | 14 | 128 | 124023 | 124151 | 191587 | 191715 | Palindromic |
|  |  | 15 | 123 | 81363 | 81486 | 200295 | 200418 | Direct |
|  |  | 16 | 111 | 210802 | 210913 | 277017 | 277128 | Direct |
|  |  | 17 | 110 | 84451 | 84561 | 274021 | 274131 | Palindromic |
|  | II | 1 | 336 | 147828 | 148164 | 197064 | 197400 | Direct |
| Somatic hybrids | I | 1 | 11916 | 34475 | 46391 | 327806 | 339722 | Palindromic |
|  |  | 2 | 11846 | 132386 | 144232 | 249413 | 261259 | Palindromic |
|  |  | 3 | 1643 | 261123 | 262766 | 345427 | 347070 | Palindromic |
|  |  | 4 | 1024 | 165998 | 167022 | 287227 | 288251 | Direct |
|  |  | 5 | 206 | 91452 | 91658 | 262359 | 262565 | Direct |
|  |  | 6 | 206 | 91452 | 91658 | 345628 | 345834 | Palindromic |
|  |  | 7 | 173 | 88104 | 88277 | 235285 | 235458 | Direct |
|  |  | 8 | 165 | 93697 | 93862 | 362978 | 363143 | Palindromic |
|  |  | 9 | 156 | 234933 | 235089 | 362997 | 363153 | Direct |
|  |  | 10 | 156 | 188956 | 189112 | 265081 | 265237 | Palindromic |
|  |  | 11 | 146 | 93697 | 93843 | 234933 | 235079 | Palindromic |
|  |  | 12 | 140 | 63895 | 64035 | 157425 | 157565 | Direct |
|  |  | 13 | 136 | 132386 | 132522 | 346934 | 347070 | Direct |
|  |  | 14 | 135 | 64096 | 64231 | 157587 | 157722 | Direct |
|  |  | 15 | 128 | 92678 | 92806 | 288114 | 288242 | Palindromic |
|  |  | 16 | 128 | 92678 | 92806 | 166885 | 167013 | Palindromic |
|  |  | 17 | 124 | 79354 | 79478 | 247237 | 247361 | Direct |
|  |  | 18 | 123 | 258179 | 258302 | 279411 | 279534 | Palindromic |
|  |  | 19 | 123 | 135343 | 135466 | 279411 | 279534 | Direct |
|  |  | 20 | 110 | 70856 | 70966 | 132268 | 132378 | Palindromic |

**Table S6**. Distribution of tandem repeats of somatic hybrids and its parental species, *S. tuberosum* and *S. commersonii*. Tandem repeats were searched using Tandem Repeats Finder program with parameters such as match 2, mismatch 7, indels 7, minimum alignment score 80, maximum period size 10, maximum TR size 50 (https://tandem.bu.edu/trf/trf.html).

| Species | Mt type | No. | Position | Consensus sequence (5′ → 3′) | Consensus size (bp) | Copy Number | Matches (%) | Location |
| --- | --- | --- | --- | --- | --- | --- | --- | --- |
| *S. tuberosum* | I | TR1 | 20082-20107 | GGATAATAGGATA | 13 | 2 | 100 | *nad1~nad5* |
|  |  | TR2 | 79413-79498 | GTATGATGAGCTTGAGTCGAGTCACAAAAAAGG | 33 | 2 | 94 | *rps4~nad4* |
|  |  | TR3 | 82108-82134 | GTAGATTCA | 9 | 3 | 100 | *rps4~nad4* |
|  |  | TR4 | 122042-122096 | CCTCTCCTTTCAGGCGAGCTACTTCGTA | 28 | 2 | 85 | *rrn26* |
|  |  | TR5 | 126111-126151 | TGATTCGTGTAG | 12 | 3 | 75 | *orf261~orfx* |
|  |  | TR6 | 126106-126160 | AGTTCTGATTCGTGTAGTGATTC | 23 | 2 | 100 | *orf261~orfx* |
|  |  | TR7 | 243363-243409 | GGAAGTGTCCGATCATAGCACGAT | 24 | 2 | 86 | *rpl16* |
|  |  | TR8 | 264985-265019 | GAATAGTCATCGCTTT | 16 | 2 | 89 | *orf104~trnM-CAU* |
|  |  | TR9 | 292534-292580 | GGAAGTGTCCGATCATAGCACGAT | 24 | 2 | 86 | *rpl16* |
|  | III | TR1 | 4013-4138 | GAGCGAGAGGAACGGACAACAGATAAAGAGTTGTAGCTAGGTCCTATAGAAATTGGAATAA | 61 | 2 | 100 | trnS-GGA~sdh4 |
|  |  | TR2 | 19487-19548 | CTAATACTAATCTAATATAATAGT | 24 | 2 | 80 | trnV-GAC~ccmFN |
|  |  | TR3 | 61785-61863 | GGGGTTAACCTGTCGATCAACTCATTTC | 28 | 2 | 96 | orf140~ccmFc |
|  |  | TR4 | 83738-83781 | CTAGTAAATAAGATATTGTTTC | 22 | 2 | 100 | orf169~orf102 |
|  |  | TR5 | 106964-107082 | GGGAGAGGGAGGCTGAAAAGGAGGCTCGACCTACA | 35 | 3 | 97 | orf123~trnD-GUC |
|  | V | TR1 | 3227-3264 | TATAAGCAACTCTTTAGTG | 19 | 2 | 89 | *orf103~ccmB* |
|  |  | TR2 | 14201-14254 | AATATAGAATAAAAGGATTTCAAGTCT | 27 | 2 | 96 | *orf127~trnE-UUC* |
|  |  | TR3 | 23411-23440 | CTTTATGATATTAAG | 15 | 2 | 100 | *orf103~sdh3* |
| *S. commersonii* | I | TR1 | 5769-5803 | TTCAAACAGATGACTA | 16 | 2 | 89 | *trnM-CAU~orf104* |
|  |  | TR2 | 27369-27415 | TCGTGCTATAAGCGGACAATTCCA | 24 | 2 | 86 | *rpl16* |
|  |  | TR3 | 49443-49546 | CTTCTCTTCAATCCACAACAACTATTAACGATT | 33 | 3 | 97 | *nad1~atp6* |
|  |  | TR4 | 86646-86689 | CTAGTAAATAAGATATTGTTTC | 22 | 2 | 100 | *orf169~orf102* |
|  |  | TR5 | 191255-191279 | ACTACTTCG | 9 | 2 | 100 | *atp6* |
|  |  | TR6 | 207521-207573 | TCCAATATGGAATAAAAGGATTTCTAC | 27 | 2 | 100 | *rpl10~rpl2* |
|  |  | TR7 | 219897-219926 | CTTTATGATATTAAG | 15 | 2 | 100 | *orf103~sdh3* |
|  |  | TR8 | 236403-236446 | TATTAGTAAGGTTGTCAAATTT | 22 | 2 | 100 | *trnH-GUG~orf109* |
|  |  | TR9 | 248966-249003 | TATAAGCAACTCTTTAGTG | 19 | 2 | 89 | *orf103~ccmB* |
|  |  | TR10 | 263350-263404 | ATAGTAGATCACCTTTTGCTTAGCTTA | 27 | 2 | 92 | *orf185~atp1* |
|  | II | TR1 | 16692-16732 | GAATCACTACAC | 12 | 3 | 75 | *orfx~orf140* |
|  |  | TR2 | 16683-16737 | ATCAGAACTGAATCACTACACGA | 23 | 2 | 100 | *orfx~orf140* |
|  |  | TR3 | 20747-20801 | ACGAAGTCACTCGACTAAAAGGAGAGGT | 28 | 2 | 85 | *rrn26* |
|  |  | TR4 | 59056-59141 | TCGACTCAAGCTCATCATACCCTTTTTGTGAC | 32 | 2 | 94 | *nad4~rps4* |
|  |  | TR5 | 138579-138603 | ACTACTTCG | 9 | 2 | 100 | *atp6* |
|  |  | TR6 | 154845-154897 | TCCAATATGGAATAAAAGGATTTCTAC | 27 | 2 | 100 | *rpl10~rpl2* |
|  |  | TR7 | 167221-167250 | CTTTATGATATTAAG | 15 | 2 | 100 | *orf103~sdh3* |
|  |  | TR8 | 183727-183770 | TATTAGTAAGGTTGTCAAATTT | 22 | 2 | 100 | *trnH-GUG~orf109* |
|  |  | TR9 | 196290-196327 | TATAAGCAACTCTTTAGTG | 19 | 2 | 89 | *orf103~ccmB* |
|  |  | TR10 | 210674-210728 | ATAGTAGATCACCTTTTGCTTAGCTTA | 27 | 2 | 92 | *orf185~atp1* |
| Somatic hybrids | I | TR1 | 6099-6184 | TCGACTCAAGCTCATCATACCCTTTTTGTGAC | 32 | 2 | 94 | *nad4~rps4* |
|  |  | TR2 | 81695-81756 | TATTAGATTAGTATTAGACTATTA | 24 | 2 | 80 | *ccmFn~trnV-GAC* |
|  |  | TR3 | 130141-130184 | GAAACAATATCTTATTTACTAG | 22 | 2 | 100 | *orf102~orf169* |
|  |  | TR4 | 143974-144051 | AGTTCTAGACTTCTCACTATCAGTC | 25 | 3 | 63 | *ccmFc~atp6* |
|  |  | TR5 | 167284-167387 | AGAAGAATCGTTAATAGTTGTTGTGGATTGAAG | 33 | 3 | 97 | *atp6* |
|  |  | TR6 | 189415-189461 | GGAAGTGTCCGATCATAGCACGAT | 24 | 2 | 86 | *rpl16* |
|  |  | TR7 | 211027-211061 | GAATAGTCATCGCTTT | 16 | 2 | 89 | *trnG-GCC~trnM-CAU* |
|  |  | TR8 | 241510-241588 | GGGGTTAACCTGTCGATCAACTCATTTC | 28 | 2 | 96 | *orf261~ccmFc* |
|  |  | TR9 | 265660-265714 | TTAAGCAAAACAAAAGGTGATCTACTA | 27 | 2 | 92 | *atp1~orf185* |
|  |  | TR10 | 288551-288575 | AAGTAGTCG | 9 | 2 | 100 | *atp6~atp6* |
|  |  | TR11 | 362174-362214 | GAATCACTACAC | 12 | 3 | 75 | *orfx~orf140* |
|  |  | TR12 | 362165-362219 | ATCAGAACTGAATCACTACACGA | 23 | 2 | 100 | *orfx~orf140* |
|  |  | TR13 | 366229-366283 | ACGAAGTCACTCGACTAAAAGGAGAGGT | 28 | 2 | 85 | *rrn26* |
|  | II | TR1 | 3232-3269 | TATAAGCAACTCTTTAGTG | 19 | 2 | 89 | *orf103~ccmB* |
|  |  | TR2 | 11023-11075 | TCCAATATGGAATAAAAGGATTTCTAC | 27 | 2 | 100 | *rpl10~rpl2* |
|  |  | TR3 | 23399-23428 | CTTTATGATATTAAG | 15 | 2 | 100 | *orf103~sdh3* |

**Table S7**. Mitochondrial gene contents of somatic hybrid, and its parental species, *S. tuberosum* and *S. commersonii* with other 10 Solanaceous plants

| Group of genes | Genes | Somatic hybrid | *Solanum tubersoum* | *Solanum tubersoum* cv. Cicero | *Solanum commersonii* | *Solanum lycopersicum* | *Solanum pennellii* | *Capsicum annum* | *Capsicum annum* (CMS) | *Hyoscyamus niger* | *Physochlaina orientalis* | *Nicotiana attenuata* | *Nicotiana sylvestris* | *Nicotiana tabacum* |
| --- | --- | --- | --- | --- | --- | --- | --- | --- | --- | --- | --- | --- | --- | --- |
|  |  | MF989958-MF989959 | MF989953-MF989957 | MN114537-MN114539 | MF989960-MF989961 | MF034192 | MF034194 | KJ865410 | KJ865409 | KM207685 | MK490961 | MF579563 | KT997964 | BA000042 |
| Complex I | *nad1* | + | + | + | + | + (newly annotated) | + | + | + | + | + | + | + | + |
|  | *nad2* | + | + | + | + | + (newly annotated) | + | + | + | + | + | + | + | + |
|  | *nad3* | + | + | + | + | + | + | + | + | + | + | + | + | + |
|  | *nad4* | + | + | + | + | + | + | + | + | + | + | + | + | + |
|  | *nad4L* | + | + | + | + | + | + | + | + | + | + | + | + | + |
|  | *nad5* | + | + | + | + | + (newly annotated) | + | + | + | + | + | + | + | + |
|  | *nad6* | + | + | + | + | + | + | + | + | + | + | + | + | + |
|  | *nad7* | + | + | + | + | + | + | + | + | + | + | + | + | + |
|  | *nad9* | + | + | + | + | + | + | + | + | + | + | + | + | + |
| Complex II | *sdh3* | + | + | + | + | + | + | + | + | + | + | + | + | + |
|  | *sdh4* | + | + | + | + | + | + | + | + | + | + | + | + (newly annotated) | + |
| Complex III | *cob* | + | + | + | + | + | + | + | + | + | + | + | + | + (orf125e) |
| Complex IV | *cox1* | + | + | + | + | + | + | + | + | + | + | + | + | + |
|  | *cox2* | + | + | + | + | + (newly annotated) | + | + | + | + | + | + | + | + |
|  | *cox3* | + | + | + | + | + | + | + | + | + | + | + | + | + |
| Complex V | *atp6* | + | + (newly annotated) | + | + | + | + | + | + | + | + | + | + | + |
|  | *atp8* | + (orfB) | + (orfB) | + | + (orfB) | + | + | + (orfB) | + (orfB) | + | + | + | + (newly annotated) | + (orfB) |
|  | *atp9* | + | + | + | + | + | + | + | + | + | + | + | + | + |
| Cytochrome c | *ccmB* | + | + | + | + | + | + | + | + | + | + | + | + | + |
|  | *ccmC* | + | + | + | + | + | + | + | + | + | + | + | + | + |
|  | *ccmFc* | + | + | + | + | + | + | + | + | + | + | + | + | + |
|  | *ccmFN* | + | + | + | + | + | + | + | + | + | + | + | + | + |
| Ribosomal proteins | *rps1* | + | + | + | + | + | + | + (orf152a) | + (orf141) | + | + | + | + (newly annotated) | + (orf216) |
|  | *rps3* | + | + | + | + | + | + | + | + | + | + | + | + | + |
|  | *rps4* | + | + | + | + | + | + | + | + | + | + | + | + | + |
|  | *rps7* | - | - | - | - | - | - | - | - | - | - | - | - | - |
|  | *rps10* | + | + | + | + | + | + | + | + | + | + | + | + | + |
|  | *rps12* | + | + | + | + | + | + | + | + | + | + | + | + | + |
|  | *rps13* | + | + | + | + | + | + | + | + | + | + | + | + | + |
|  | *rps14* | - (pseudo) | - (pseudo) | - (pseudo) | - (pseudo) | - (pseudo) | - (pseudo) | - (pseudo) | - (pseudo) | - (pseudo) | - (pseudo) | + | + | + |
|  | *rps19* | + | + | + | + | + | + | + | + | + | + | + | + | + |
|  | *rpl2* | + | + | + | + | + | + | + | + | + | + | + | + | + |
|  | *rpl5* | + | + | + | + | + | + | + | + | + | + | + | + | + |
|  | *rpl10* | + | + | + | + | + | + | + | + | + | + | + | + (newly annotated) | + (orf159b) |
|  | *rpl16* | + | + | + | + | + | + | + | + | + | + | + | + | + |
| Maturase | *matR* | + | + | + | + | + | + | + | + | + | + | + | + | + |
| Transferase | *mttB* | + (orfX) | + (orfX) | + | + (orfX) | + | + | + (orfX) | + (orfX) | + | + | + | + (newly annotated) | + (orfX) |

**Table S8**. Ks, Ka, and Ks/Ka values of mitogenome genes in 13 species of Solanaceous family

| Gene | Ka | Ks | Ka/Ks |
| --- | --- | --- | --- |
| *nad1* | 0.000605 | 0.0034 | 0.177807 |
| *nad2* | 0.00015 | 0.001732 | 0.086614 |
| *nad3* | 0.002724 | 0.010256 | 0.265623 |
| *nad4* | 0 | 0.00182 | 0 |
| *nad4L* | 0 | 0.002661 | 0 |
| *nad5* | 0.00203 | 0.004723 | 0.429901 |
| *nad6* | 0.002726 | 0.022226 | 0.12264 |
| *nad7* | 0.000798 | 0.009426 | 0.084713 |
| *nad9* | 0.002885 | 0.008224 | 0.350774 |
| *sdh3* | 0.018436 | 0.025944 | 0.710623 |
| *sdh4* | 0.002524 | 0.01663 | 0.151786 |
| *cob* | 0.000648 | 0.017161 | 0.037789 |
| *cox1* | 0.000727 | 0.013552 | 0.053667 |
| *cox2* | 0.003402 | 0.016462 | 0.206627 |
| *cox3* | 0.000686 | 0.003018 | 0.22741 |
| *atp1* | 0.001735 | 0.024229 | 0.071603 |
| *atp4* | 0.002764 | 0.004153 | 0.665451 |
| *atp6* | 0.118573 | 0.227817 | 0.520474 |
| *atp8* | 0.004267 | 0.00798 | 0.53465 |
| *atp9* | 0.001909 | 0.002644 | 0.722063 |
| *ccmB* | 0.001009 | 0.002891 | 0.349057 |
| *ccmC* | 0.020385 | 0.028959 | 0.703919 |
| *ccmFc* | 0.003612 | 0.003523 | 1.025376 |
| *ccmFN* | 0.008582 | 0.012708 | 0.675331 |
| *rps3* | 0.003433 | 0.007444 | 0.461225 |
| *rps4* | 0.001839 | 0.014764 | 0.124589 |
| *rps10* | 0.00687 | 0.01348 | 0.50961 |
| *rps12* | 0.000567 | 0.005936 | 0.095457 |
| *rps13* | 0.003527 | 0.020055 | 0.175884 |
| *rpl2* | 0.008308 | 0.009895 | 0.839535 |
| *rpl5* | 0.007471 | 0.026117 | 0.286071 |
| *rpl10* | 0.00322 | 0.016574 | 0.194259 |
| *rpl16* | 0.004826 | 0.011405 | 0.423143 |
| *matR* | 0.006611 | 0.009624 | 0.68687 |
| *mttB* | 0.005367 | 0.001521 | 3.527888 |

**Table S9**. Best model evaluation of nucleotide sequence evolution by jModelTest (ver. 2.1.10)

|  | Model | f(a) | f(c) | f(g) | f(t) | kappa | titv | Ra | Rb | Rc | Rd | Re | Rf | pInv | gamma |
| --- | --- | --- | --- | --- | --- | --- | --- | --- | --- | --- | --- | --- | --- | --- | --- |
| AIC | GTR+I+G | 0.270 | 0.210 | 0.220 | 0.310 | 0.000 | 0.000 | 1.806 | 2.430 | 0.628 | 1.298 | 2.431 | 1.000 | 0.860 | 0.800 |
| BIC | GTR+I+G | 0.270 | 0.210 | 0.220 | 0.310 | 0.000 | 0.000 | 1.806 | 2.430 | 0.628 | 1.298 | 2.431 | 1.000 | 0.860 | 0.800 |

**Table S10**. Distribution of SNP and Indel variations on 45S nrDNA sequences among three species.

| **No.** | **45S rDNA position of Somatic hybrid** | ***S. tuberosum*** | ***S. commersonii*** | ***Somatic hybrid*** | **Variation type** |
| --- | --- | --- | --- | --- | --- |
| 1 | 191 | T | C | C/T | SNP |
| 2 | 1814 | A | C | A/C | SNP |
| 3 | 1819 | C | CA | C/CA | InDel |
| 4 | 1821 | G | A | A/G | SNP |
| 5 | 1846 | TT | TTA | TT/TTA | InDel |
| 6 | 1858 | GG | GGGGCGACGCGCGCCGGTC | GG/GGGGCGACGCGCGCCGGTC | InDel |
| 7 | 1863 | CTTGCGC | CGCCTCC | CTTGCGC/CGCCTCC | InDel |
| 8 | 1883 | A | G | A/G | SNP |
| 9 | 1884 | CGACTTGC | CCT | CCT/CGACTTGC | InDel |
| 10 | 1895 | CTT | CGA | CGA/CTT | InDel |
| 11 | 1900 | G | T | G/T | SNP |
| 12 | 1905 | CGTTTTTG | C | C/CGTTTTTG | InDel |
| 13 | 1915 | GGCCAA | GGA | GGA/GGCCAA | InDel |
| 14 | 1922 | G | C | C/G | SNP |
| 15 | 1971 | CT | CAC | CT/CAC | InDel |
| 16 | 1977 | TCGCGCCCCGTTCGCGGATCGCCCG | TT | TT/TCGCGCCCCGTTCGCGGATCGCCCG | InDel |
| 17 | 2029 | A | G | A/G | SNP |
| 18 | 2209 | G | A | A/G | SNP |
| 19 | 2211 | A | G | A/G | SNP |
| 20 | 2212 | CCTT | CCGCAAGGCGCG | CCTT/CCGCAAGGCGCG | InDel |
| 21 | 2313 | A | G | A/G | SNP |
| 22 | 2318 | A | G | A/G | SNP |
| 23 | 2351 | C | T | C/T | SNP |
| 24 | 2355 | T | C | C/T | SNP |
| 25 | 2363 | T | C | C/T | SNP |
| 26 | 2380 | CCATG | CGCCTCCGTGC | CCATG/CGCCTCCGTGC | InDel |
| 27 | 2526 | T | C | C/T | SNP |
| 28 | 2859 | T | C | C/T | SNP |
| 29 | 2960 | T | C | C | SNP |
| 30 | 2986 | C | C | C/T | SNP |
| 31 | 3139 | C | C | C/T | SNP |
| 32 | 3763 | A | G | A/G | SNP |
| 33 | 3811 | T | C | C/T | SNP |

# **Supplementary Figures**

**
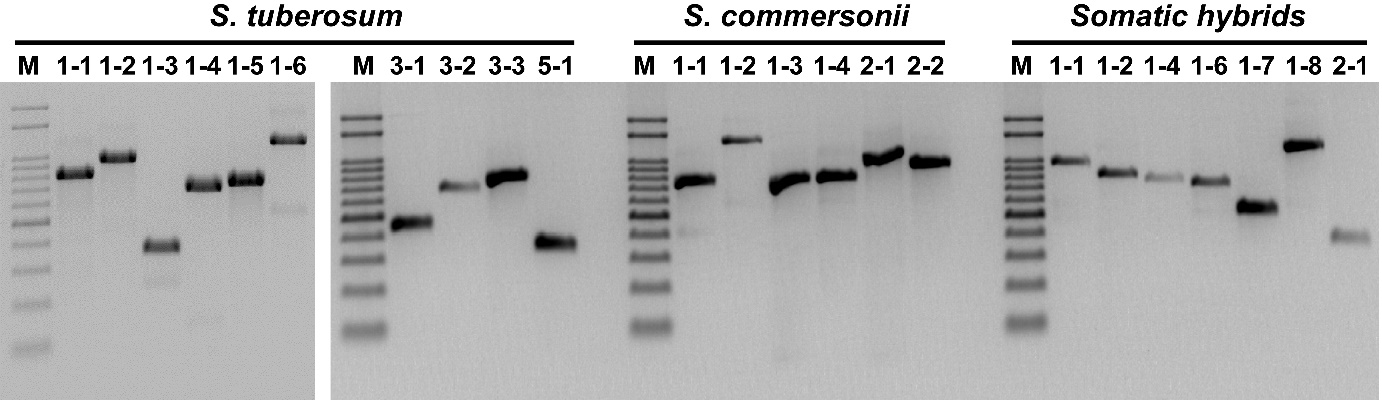
**

**Figure S1**. Validation of complete mitogenome sequence using type-specific PCR primers. The detailed mt type specific PCR primers information was shown in (Table S2). The agarose gel electrophoresis divided into left and right was a different agarose gel and the uncropped images of Figure S1 as follows. The red rectangle indicates the relevant areas of the images shown in Figure S1.


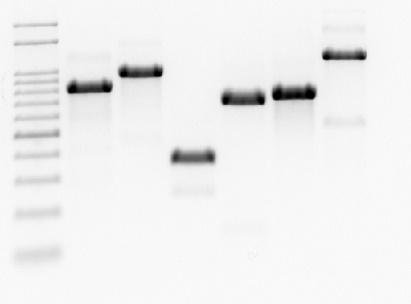


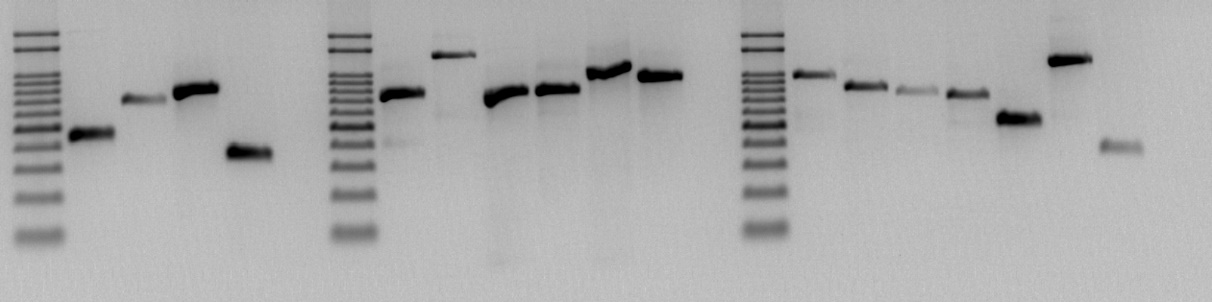


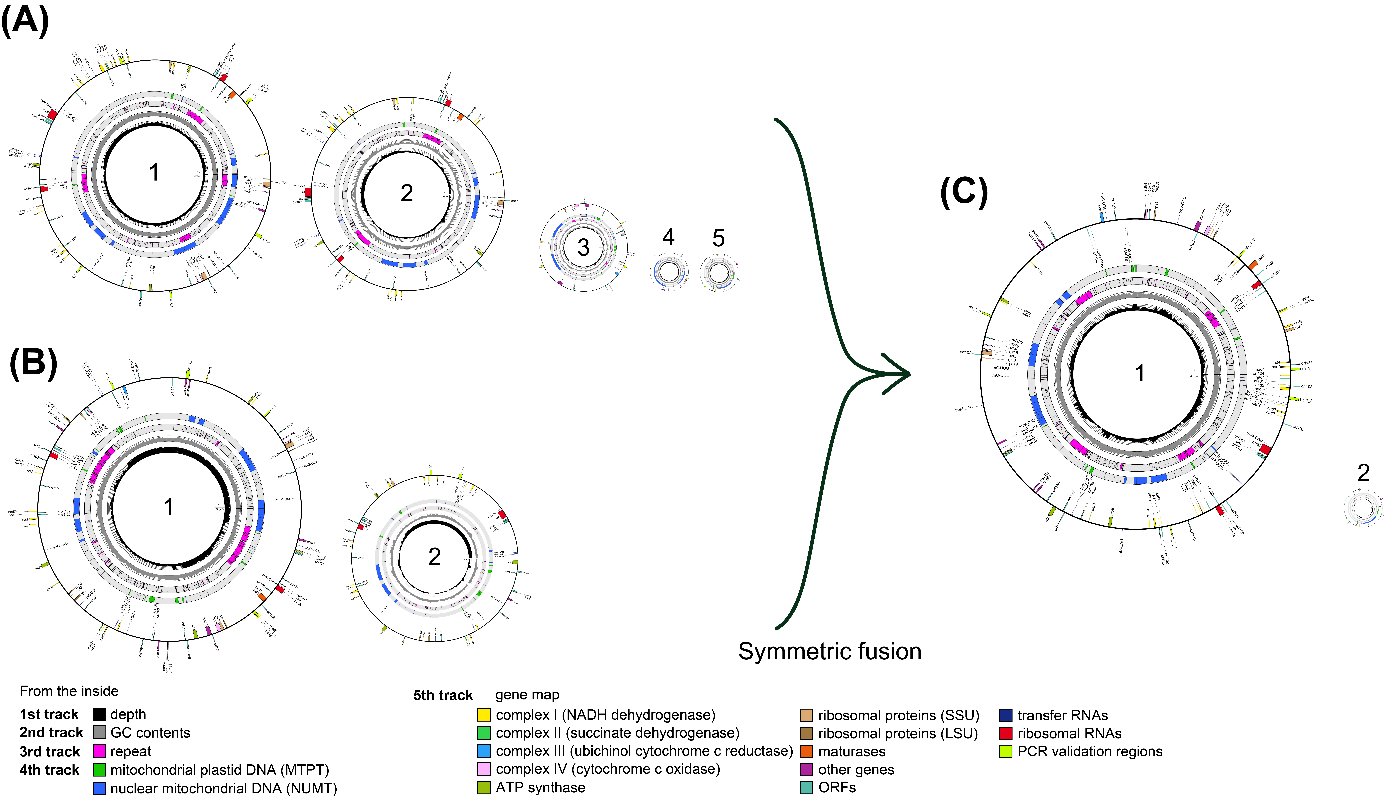


**Figure S2.** The circular mitogenome maps. (A) *Solanum tuberosum,* (B) *S. commersonii,* and (C) somatic hybrids between *S. tuberosum* and *S. commersonii*. The first track (innermost circle) indicates mapping depth of PE reads (each grey circle 20x, maximum 100x); The second track indicates GC contents (each grey circle 20%, maximum 100%, window size 100 bp); The third track indicates the distribution of repeats (Tables S5 and S6); the fourth track indicates sequences considered to be derived from chloroplast (green) and nuclear genome (blue)(Table 1); the fifth track (outmost circle) indicates position of predicted genes (protein-coding, rRNA and tRNA genes). The positions of PCR primers used for validation are marked as a green fluorescent color.


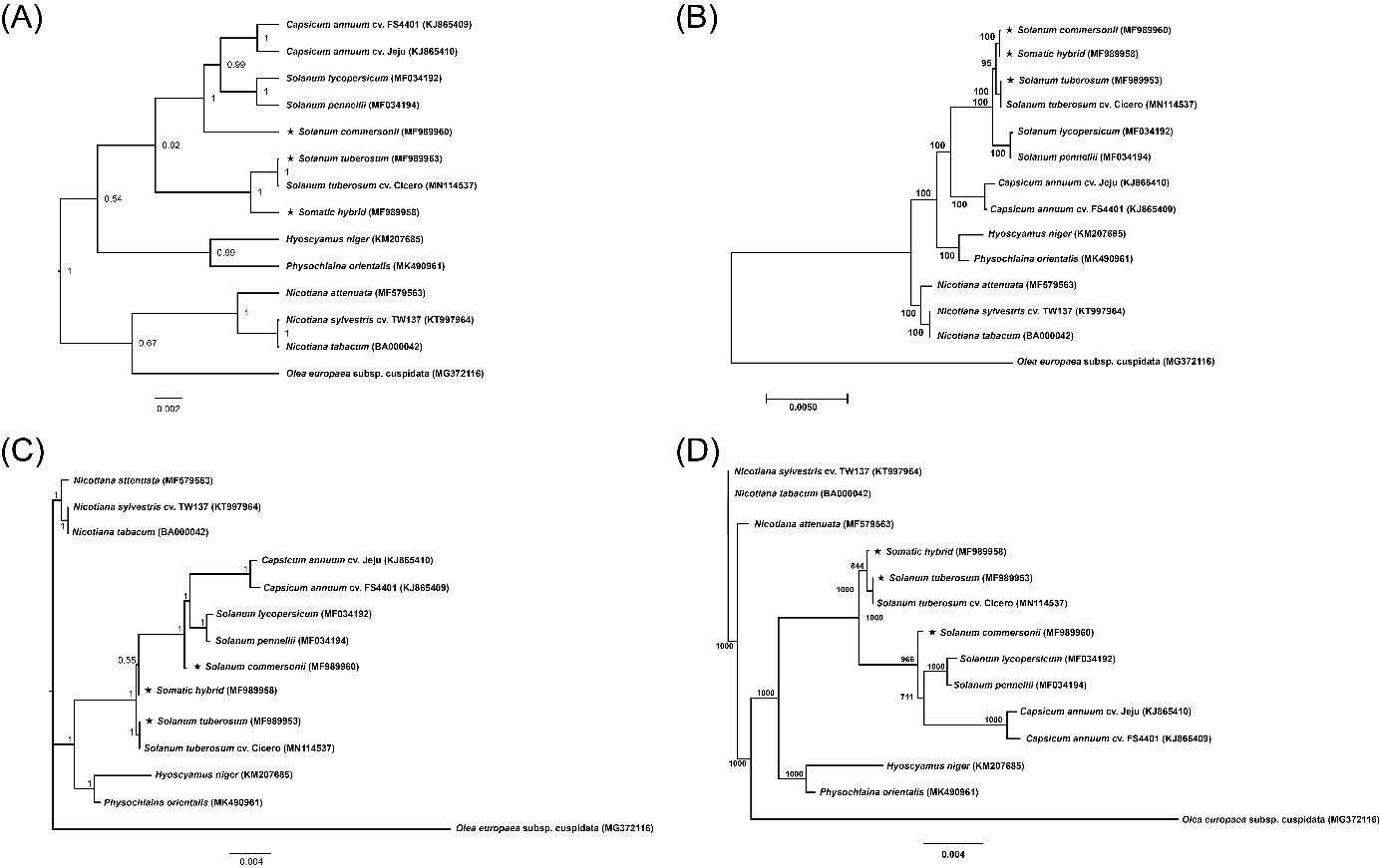


**Figure S3**. Bayesian and maximum likelihood (ML) phylogenetic trees of 13 Solanaceae species and 1 Oleaceae species based on commonly conserved 35 protein-coding sequences in mitogenomes. The bootstrap and posterior probability value were on the node. (A) Bayesian phylogenetic tree was constructed using BEAST program (ver. 2.6.2) with general time reversible (GTR) + gamma (Γ) nucleotide substitution model, relaxed clock log normal strict model, and 10 million MCMC chain length options. (B) ML phylogenetic tree was constructed using MEGA7 program with GTR + Γ + I (invariable) nucleotide substitution model and 1,000 bootstrapping options. (C) Bayesian phylogenetic tree was constructed using MrBayes with nucleotide model= 4by4, Nst = 6, inverse gamma (invgamma) distribution for likelihood model, and 5 million MCMC chain length options. (D) ML phylogenetic tree was constructed using PhyML (ver. 3.0) with GTR nucleotide substitution model and 1,000 bootstrapping options.


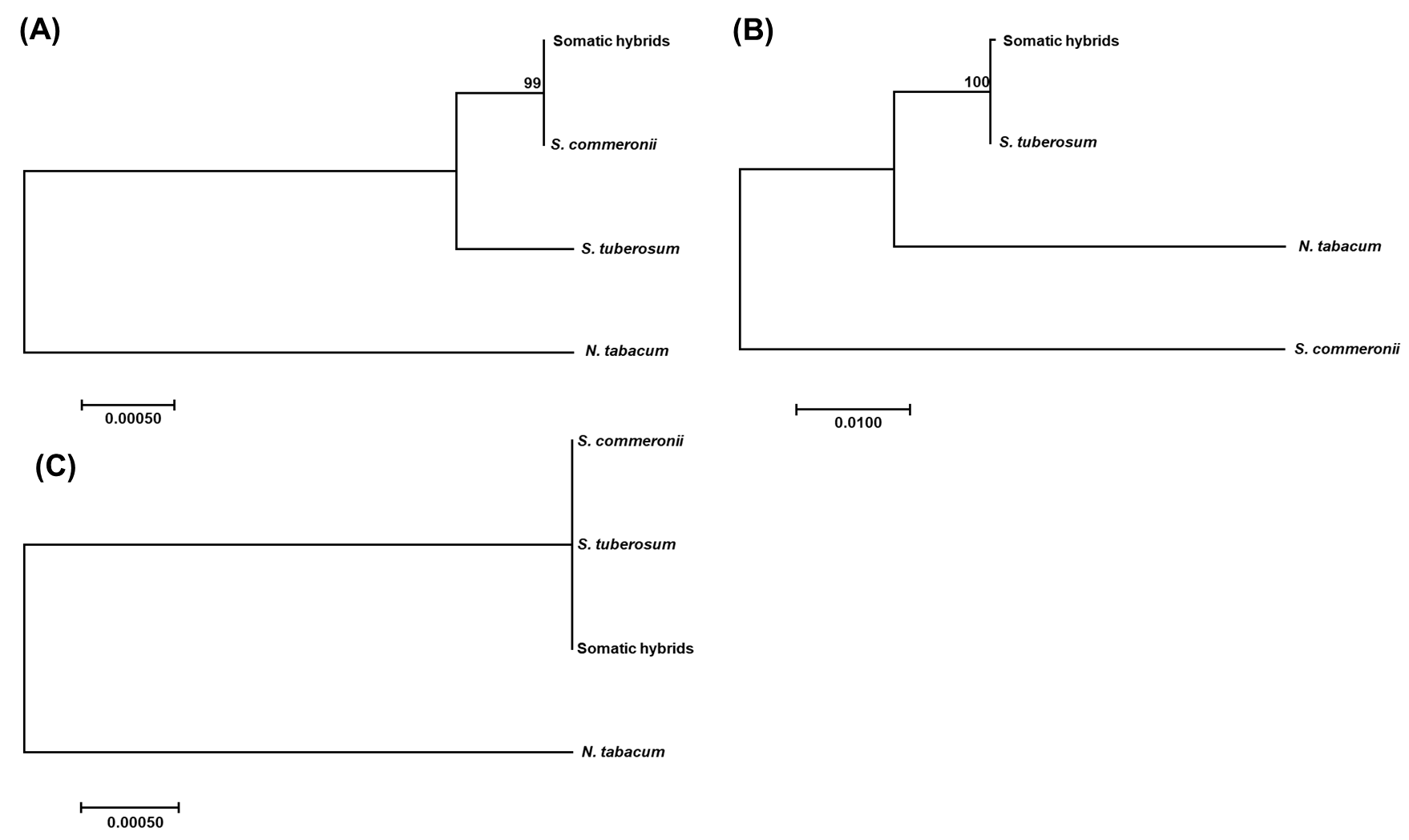


**Figure S4**. Maximum likelihood phylogenetic tree of somatic hybrid, and its parental species, *S. tuberosum* and *S. commersonii* using protein coding genes (CDS)*.* (A) tree based on 12 CDSs originated from *S. commeronii* (*nad1, nad2, nad7, nad9, sdh4, cob, cox1, ccmC, ccmFc, rps10, rpl2, matR*). (B) tree based on 2 CDSs originated from *S. tuberosum* (*atp6, ccmFN*). (C) tree based on 21 CDSs undistinguishable (*nad3, nad4, nad4L, nad5, nad6, sdh3, cox2, cox3, atp1, atp4, atp8, atp9, ccmB, rps3, rps4, rps12, rps13, rpl5, rpl10, rpl16, mttB*).

**
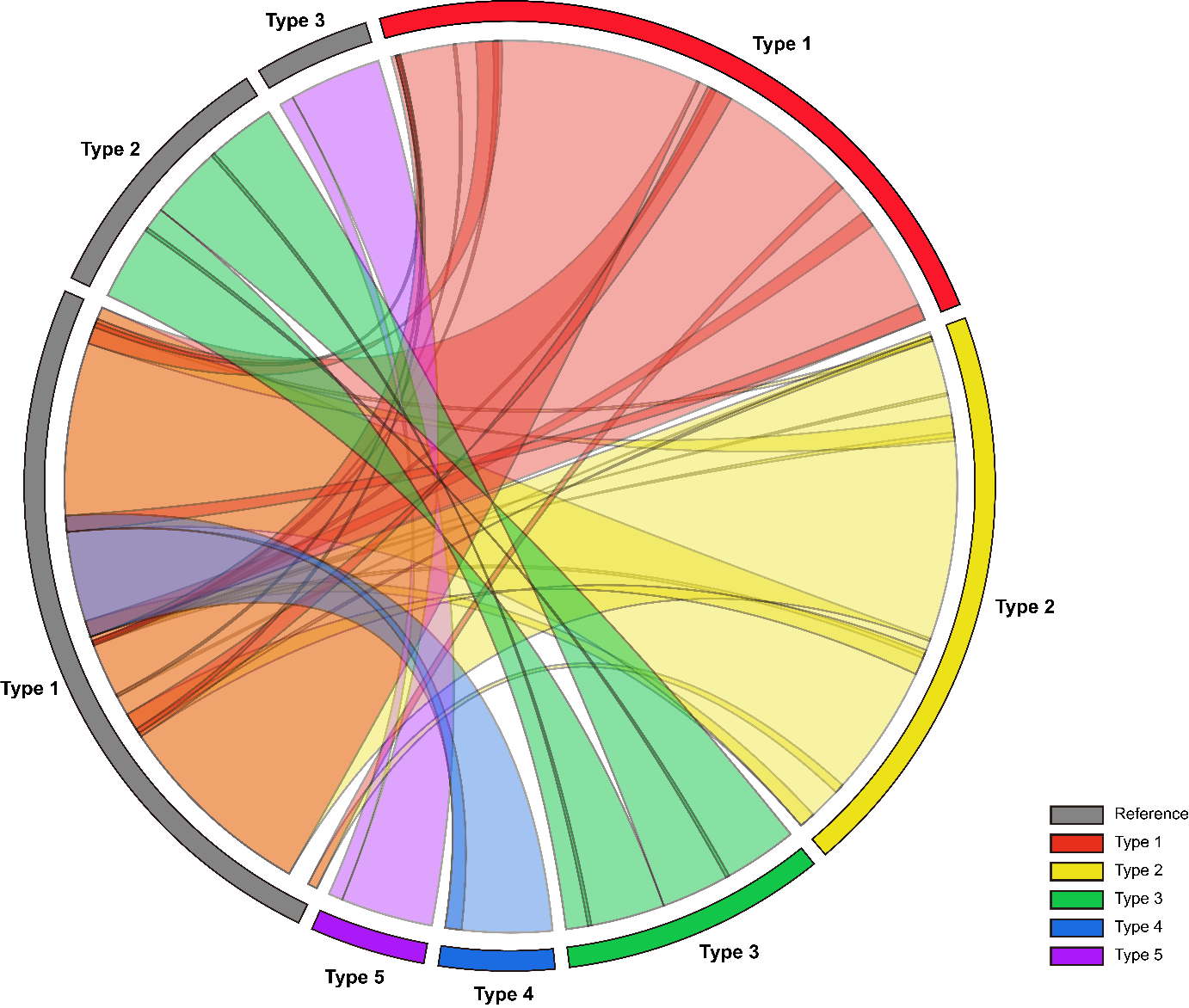
Figure S5**. Chord diagram of *Solanum tuberosum* mitogenome homology at intra-species level (*S. tuberosum* vs. *S. tuberosum* cv. Cicero). Diagram was drawn by circos R package. Genome synteny were identified by BLASTN search (cutoff E-value 1E-1, minimum match length 1,000 bp).
